# Adjacent-Site Proximity as a Dominant Activity Descriptor in Single-Atom Pt Catalysts for Hydrogen Evolution Reaction

**DOI:** 10.1007/s40820-026-02201-z

**Published:** 2026-05-04

**Authors:** Xue-Lu Chen, Yu-Yang Liu, Sudip Biswas, Yi Yang, Yi Shi, Chun-Gen Liu, Xing-Hua Xia

**Affiliations:** 1 State Key Laboratory of Analytical Chemistry for Life Science, School of Chemistry and Chemical Engineering, Nanjing University, Nanjing, 210023 People’s Republic of China; 2 Institute of Theoretical and Computational Chemistry, School of Chemistry and Chemical Engineering, Nanjing University, Nanjing, 210023 People’s Republic of China; 3 School of Chemistry and Molecular Engineering, East China Normal University, Dongchuan Road 500, Shanghai, 200241 People’s Republic of China

**Keywords:** Single-atom platinum, Interatomic proximity, H–adsorption mode, Hydrogen evolution reaction, Pt–H–Pt intermediate

## Abstract

Precise control of adjacent-site proximity and electronic states in single-atom catalysts (SACs) enable atomic-level modulation of intrinsic catalytic properties. While the influence of electronic structure on catalytic performance is well established, the impact of adjacent-site proximity remains underexplored. Here, we report the single-atom platinum catalysts on MoS_2_ (Pt-SAC/MoS_2_), in which both the controlled enrichment of adjacent Pt (Pt_adj_) sites and the Pt oxidation state are tuned via galvanic displacement of underpotentially deposited Cu adatoms. We find that hydrogen evolution reaction (HER) activity is predominantly governed by non-bonded Pt∙∙∙Pt proximity rather than oxidation state: enriched Pt_adj_ sites in Pt_SA_-0.1/MoS_2_ exhibits a mass activity 41-fold higher than isolated Pt (Pt_iso_) sites in Pt_SA_-0.3/MoS_2_ under acidic conditions. In situ infrared spectroscopy reveals that Pt_iso_ sites preferentially bind linear adsorbed hydrogen intermediate (^*^H_L_), whereas Pt_adj_ sites stabilize bridge hydrogen intermediate (^*^H_B_), which is indicative of adjacent-site proximity. Density functional theory calculations reveal that neighboring Pt atoms promote the formation of a three-center “Pt–H–Pt” bonding intermediate, which lowers the H–H coupling barrier and accelerates HER kinetics. These findings establish adjacent-site proximity as a dominant activity descriptor in SACs and provide new design principles for next-generation high-performance electrocatalysts.

**Supplementary Information:**

The online version contains supplementary material available at 10.1007/s40820-026-02201-z.

## Highlights

- Site-specific underpotential deposition strategy enables precise spatial control over atomic-level active sites, enriching adjacent Pt sites while tuning oxidation states
- Adjacent-site proximity dominates hydrogen evolution reaction (HER) activity, with adjacent Pt sites deliver a 41-fold higher mass activity than isolated Pt sites.
- Pt–H–Pt bridge intermediate at adjacent sites lowers the H–H coupling barrier and accelerates HER kinetics.

**Supplementary Information:**

The online version contains supplementary material available at 10.1007/s40820-026-02201-z.

## Graphical abstract

**Figure:**
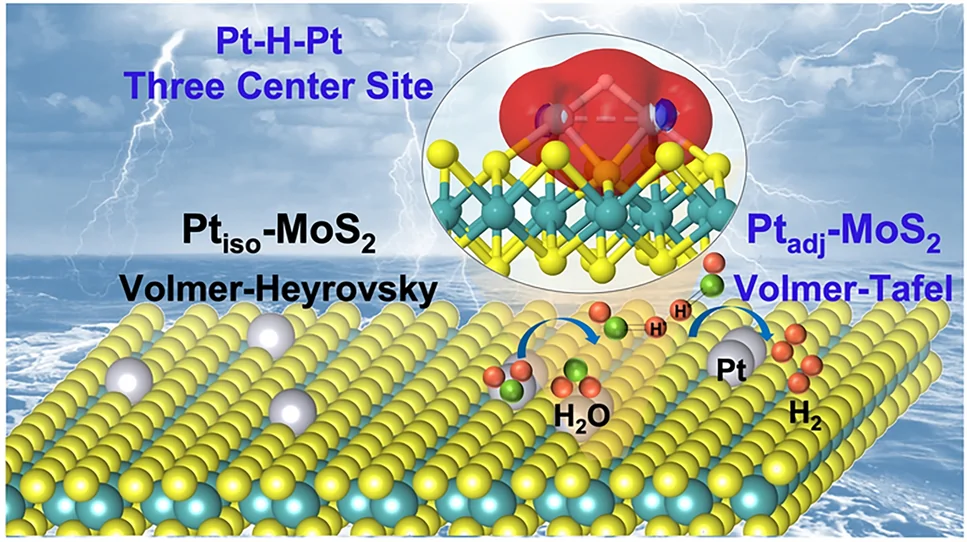

## Introduction

Green hydrogen represents the future of sustainable fuel and serves as a pivotal medium for large-scale energy storage and conversion. Among the available production routes, the electrocatalytic hydrogen evolution reaction (HER), powered by renewable electricity, offers a practical and scalable pathway to carbon-free hydrogen [1–4]. However, the kinetic barriers of water splitting necessitate highly efficient electrocatalysts [5, 6]. An efficient HER electrocatalyst must optimize hydrogen adsorption energy with the kinetics of atomic hydrogen formation and desorption [7]. Platinum remains the benchmark HER catalyst due to its near-optimal hydrogen adsorption energy and exceptional intrinsic activity [8–11]. However, its scarcity and high cost necessitate strategies that maximize atomic utilization while enhancing catalytic performance [4, 12].

Single-atom catalysts (SACs) have emerged as a compelling solution, offering atom-level dispersion of active metals to achieve maximal utilization, and well-defined active sites [13–19]. Most SACs design strategies focus on electronic structure modulation [20, 21], while the effect of the adjacent-site proximity remains in its nascent stage of exploration. Recent studies show that, beyond oxidation state tuning via metal–support interactions, the proximity of adjacent single atom can profoundly influence reaction kinetics by lowering activation barriers and enabling cooperative catalytic mechanisms [22]. For example, tailoring the oxidation states of Pt SACs through electronic metal–support interactions (EMSI) markedly alters HER activity [23], while constructing neighboring heteronuclear sites such as (Pt–O_x_)–(Co–O_y_) can enhance both HER and oxygen reduction by reshaping reaction pathways [24]. Similarly, optimizing inter-site distances between Cu–N_4_ moieties to match specific reactant dimensions promotes Fenton-like reactions [25].

However, achieving angstrom-level structural precision remains challenging due to the inherent tendency of metal atoms to agglomerate during synthesis. Choice of a suitable support material and synthesis strategy is critical for the successful fabrication of SACs. Previous studies show that two-dimensional molybdenum disulfide (2D MoS_2_), with its isolated and non-uniformly distributed sulfur sites, effectively anchors single metal atoms owing to its Lewis basicity and favorable electronegativity [26]. The site-specific underpotential deposition (UPD) technique enables self-limiting growth, which ensures precise spatial control, along with rapid, minute-scale synthesis, and mild processing conditions, as demonstrated in our previous work [27, 28].

Herein, we report a galvanic displacement strategy based on site-specific UPD that enables simultaneous control over both the relative enrichment of adjacent Pt (Pt_adj_) sites and the electronic state of single platinum atoms on 2D MoS_2_ nanosheets. By varying the deposition potential, we prepared a series of catalysts transitioning from the isolated Pt (Pt_iso_) sites to those enriched with non-bonded Pt_adj_ sites as confirmed by atomic resolved scanning electron microscopy and X-ray absorption spectroscopy. In situ infrared spectroscopy and density functional theory (DFT) calculations reveal that the Pt_adj_ sites stabilize bridge-H adsorption geometry, while the Pt_iso_ sites can only bind atop H. The bridge-H configuration corresponds to a three-center Pt–H–Pt intermediate optimizes hydrogen adsorption energetics and accelerates HER kinetics. This work establishes adjacent-site proximity as a dominant activity descriptor for HER and provides a mechanistic blueprint for the rational design of next-generation high-performance electrocatalysts.

## Experimental Section

### Materials

Molybdenum (IV) sulfide (MoS_2_) powder, tungsten (IV) sulfide (WS_2_) powder, molybdenum (IV) selenide (MoSe_2_) powder, n-butyl lithium (2.5 M in hexanes), copper (II) sulfate pentahydrate (CuSO_4_·5H_2_O), potassium platinum (II) chloride (K_2_PtCl_4_), Pt/C (20 wt%), and Nafion perfluorinated resin solution (5 wt% in a mixture of low aliphatic alcohols and water, contains 45% water) were purchased from Sigma-Aldrich (USA). Tungsten (IV) selenide (WSe_2_) powder was purchased from Aladdin Industrial Corporation (Shanghai, China). All aqueous solutions were prepared with Millipore water (resistivity of 18.2 MΩ cm).

### Synthesis of Chemically Exfoliated Transition Metal Dichalcogenides (ce-TMDs) Nanosheets

In a typical synthesis, 300 mg pristine TMDs powder (MoS_2_, WS_2_, MoSe_2_, or WSe_2_) was initially kept at 70 °C in argon atmosphere Schlenk flask, then 5 mL n-butyl lithium in hexanes (2.5 M) solution was injected into the flask and kept at 70 °C for 48 h. Afterward, the cooled solid was washed by n-hexane (SuperDry, 97.5%, J&Kseal, water: ≤ 50 ppm) for four times to remove the lithium, then immediately transferred in water and fully ultrasonicated for 1 h. The homogeneous mixture was further dialyzed for 5 days (MWCO = 14,000 D). The suspension was then centrifuged to remove the unexfoliated materials. Finally, the ce-TMDs supernatants (ce-MoS_2_, ce-WS_2_, ce-MoSe_2_, or ce-WSe_2_) were obtained and freshly used.

### Synthesis of Pt_SA_-X/TMDs

The site-specific electrodeposition (SSED) device was performed in a U-type electrochemical cell with a proton-exchange membrane. To avoid external contamination, the Nafion 117 proton-exchange membrane was pretreated by heating in 5% H_2_O_2_ aqueous solution at 80 °C, deionized water at 80 °C for 1 h, followed by washing with 0.5 M H_2_SO_4_ at 80 °C and again deionized water at 80 °C for per one hour. In this typical three-electrode configuration, Ag/AgCl (saturated KCl) was used as the reference electrode, graphite rod or carbon paper was used as the working electrode. The half-reaction cell was filled with an argon-saturated solution of 0.1 M H_2_SO_4_ + 2 mM CuSO_4_ and ce-TMDs. A Pt wire was used as the counter electrode in the other half-reaction electrochemical cell with a solution of 0.1 M H_2_SO_4_. A constant potential (0–0.3 V *vs.* Ag/AgCl) was applied at the graphite rod or carbon paper under continuously stirring, during which a fluffy black powder was gradually adsorbed onto the working electrode. Then, a solution of 0.1 M H_2_SO_4_ containing 5 mM K_2_PtCl_4_ was dropwise added into the above solution at ice-bath temperature under continuously stirring for 30 min. Finally, the resulting product was washed several times with water and collected by centrifugation, followed by lyophilized overnight to obtain the resulting product.

### Physical Characterization

Transmission electron microscopy (TEM, JEOL JEM-2100, Japan) and field-emission high resolution transmission electron microscope (HR-TEM, Talos F200X, Thermo Fisher Scientific, USA) were utilized to characterize the morphologies and elemental maps of all samples. Atomic force microscopy (AFM) measurements were carried out using a commercial AFM (Bruker, Dimension FastScan, Icon Scanner, USA). Raman spectra were collected on a LabRAM Aramis Raman spectrometer (HORIBA, Ltd., Japan). Aberration-corrected high-angle annular dark field scanning transmission electron microscopy (HAADF-STEM) experiments were conducted on a JEM-ARM200F instrument with an accelerating energy of 200 keV, and digital micrograph software was utilized for collecting the HAADF-STEM images with atomic resolution. The sample was drop-dryed on polymer-coated Cu-grid. The crystal structure of the materials was determined using a D8 ADVANCE X-ray powder diffractometer (XRD, Bruker, Germany). Inductively coupled plasma optical emission spectrometry (ICP-OES) was used to determine the loading of single-atom Pt in Pt_SA_-X/TMDs on a CHN-ORapid (German). The samples for ICP analysis were treated in Teflon-lined autoclaves at 180 °C for 12 h. Element binding energy data were obtained using a Nexsa X-ray photoelectron spectrometer (XPS, Thermo Fisher Scientific, USA), with the C 1* s* peak at 284.8 V used as the charge correction reference. The X-ray absorption spectroscopy at the Pt *L*_3_-edge was collected at beamline BL14B2 of the Japanese Spring8 synchrotron radiation source, using a cryogenically cooled double crystal Si (111) monochromator. The data collection was carried out in transmission mode using ionization chamber for Pt foil, PtO_2_, and in transmission mode using a Lytle detector for Pt_SA_-0.1/MoS_2_, Pt_SA_-0.14/MoS_2_, Pt_SA_-0.18/MoS_2_, and in fluorescence excitation mode using a Lytle detector for Pt_SA_-0.24/MoS_2_ and Pt_SA_-0.3/MoS_2_. All spectra were collected under ambient conditions. The X-ray absorption fine structure (XAFS) data were processed according to the standard procedures using the Athena module implemented in the IFEFFIT software packages. Extended X-ray absorption fine structure (EXAFS) spectra were obtained by subtracting the post-edge background from the overall absorption and then normalizing with respect to the edge jump step. Subsequently, the *χ(k)* data were Fourier transformed to real (*R*) space using a hanning windows (*dk* = 1.0 Å^−1^) to separate the EXAFS contributions from different coordination shells. To obtain the quantitative structural parameters around central atoms, least-squares curve parameter fitting was performed using the ARTEMIS module of IFEFFIT software packages. In situ attenuated total reflectance-Fourier transform infrared (ATR-FTIR) spectroscopy measurements using a Nicolet iS50 FTIR spectrometer equipped with an MCT detector cooled with liquid nitrogen. The Pt_SA_-0.1/MoS_2_ and Pt_SA_-0.3/MoS_2_ catalysts were prepared on the ZnSe crystal as the infrared transmission window. The IR spectra were recorded at a spectral resolution of 4 cm^−1^, and 256 scans were integral to each spectrum. All spectra were presented in absorbance and defined as A =  − log(R/R_ref_), where R and R_ref_ represent the reflected intensity of the sample and reference single beam spectrum, respectively.

### Electrochemical Measurements for HER

Linear sweep voltammetry (LSV) was carried out at a scan rate of 20 mV s^−1^ using a CHI 660E electrochemical workstation (Chenhua, China). The tests were conducted in 0.5 M H_2_SO_4_ electrolyte with a three-electrode configuration: an Ag/AgCl electrode (saturated KCl) as the reference electrode, a graphite rod as the counter electrode, and a glassy carbon electrode as the working electrode. The Ag/AgCl electrode was calibrated against a reversible hydrogen electrode (RHE) according to the relationship *E*_RHE_ = *E*_Ag/AgCl_ + 0.2220 V in 0.5 M H_2_SO_4_. Electrochemical impedance spectroscopy (EIS) measurements were conducted in the frequency range of 0.1 Hz to 100 kHz with an alternating current (AC) amplitude of 10 mV, applied at the onset potential corresponding to a current density of 0.5 mA cm^−2^ for each electrocatalyst.

### ATR-FTIR Experiment

The FTIR spectra were collected during electrocatalysis at different applied potentials in an Ar-saturated 0.5 M H_2_SO_4_ solution. Pt electrode and an Ag/AgCl electrode were used as the counter and the reference electrodes, respectively. The working electrode was fabricated by drop-casting catalysts onto underlayer Au film on the ZnSe ATR hemisphere with a catalyst loading of 0.4 mg cm^−2^.

## Results and Discussion

### Rational Design of Pt_SA_-X/MoS_2_ with Controllable Interatomic Pt Spacing

**Fig. 1:**
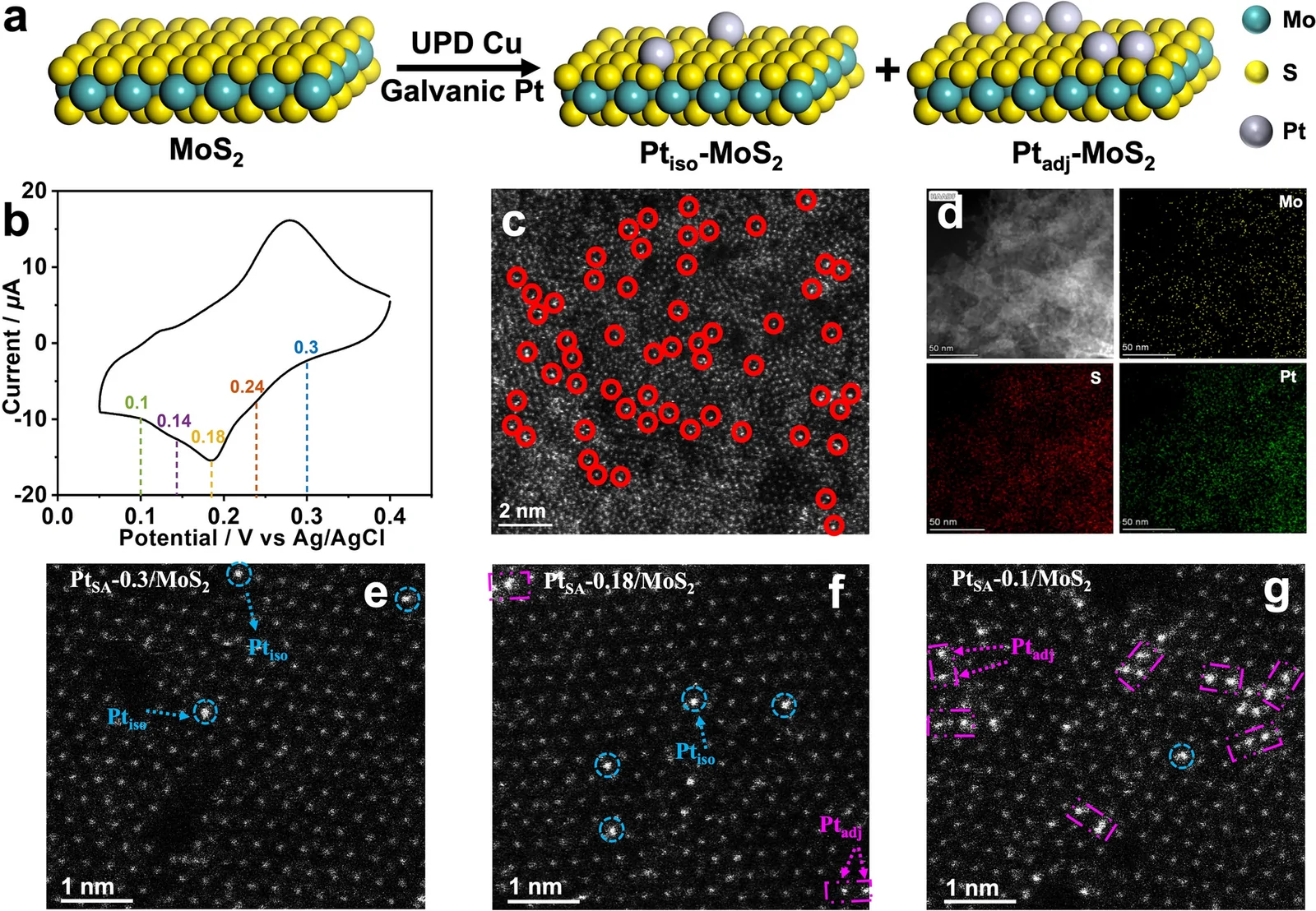
Synthesis procedure and morphological characterizations of Pt_SA_-X/MoS_2_. **a** Schematic illustration for preparing Pt_SA_-MoS_2_. **b** CV of ce-MoS_2_ modified glassy carbon electrode in an Ar-saturated solution of 0.1 M H_2_SO_4_ + 2 mM CuSO_4_ at a scan rate of 50 mV s^−1^, and five notable cathodic potentials denoted in the CV correspond to the deposition potentials for Cu adatoms in the synthesis of Cu_SA_-MoS_2_. **c** HAADF-STEM image of Pt_SA_-0.1/MoS_2_, with red circles highlighting the single atoms of Pt distributed in MoS_2_. **d** Elemental mappings of Pt_SA_-0.1/MoS_2_. HAADF-STEM images of **e** Pt_SA_-0.3/MoS_2_, **f** Pt_SA_-0.18/MoS_2_, and **g** Pt_SA_-0.1/MoS_2_. Inset, Pt_iso_ sites and Pt_adj_ sites are respectively represented in green circles and yellow rectangles

We synthesized a series of single-atom Pt catalysts (denoted Pt_SA_-X/MoS_2_, where X represents the deposition potential) using a site-specific UPD-galvanic displacement strategy (Fig. 1a). This approach enables precise control over non-bonded Pt∙∙∙Pt proximity by tuning the potential-dependent enrichment of Pt_adj_ sites on chemically exfoliated MoS_2_ nanosheet (ce-MoS_2_). By varying the deposition potential, we obtained catalysts ranging from purely Pt_iso_ sites in Pt_SA_-0.3/MoS_2_ to non-bonded Pt_adj_-enriched configurations in Pt_SA_-0.1/MoS_2_. Few-layer MoS_2_ nanosheets were prepared by organolithium-assisted exfoliation (details are given in Supplementary Materials and Methods) [29]. TEM and AFM images of ce-MoS_2_ reveal translucent, stacked nanosheets with few-layer morphology and thickness below 1 nm, in contrast to the highly overlapped, folded sheets of bulk MoS_2_ powder (Fig. S1a-e). The Raman spectra of ce-MoS_2_ display characteristic 2H-phase in-plane (E^1^_2g_) and out-of-plane (A_1g_) modes, along with additional low-frequency peaks indicative of partial 1T-phase formation upon exfoliation (Fig. S1f) [30].

The cyclic voltammogram (CV) of a ce-MoS_2_-modified electrode in Ar-saturated 0.1 M H_2_SO_4_ containing 2 mM CuSO_4_ exhibits a pronounced cathodic peak near + 0.2 V (*vs* Ag/AgCl), characteristic of underpotential deposition of Cu adatoms (Fig. S2b) [28, 31, 32]. To modulate Pt_adj_ enrichment, Cu UPD was performed at five selected deposition potentials (0.1, 0.14, 0.18, 0.24, and 0.3 V *vs.* Ag/AgCl) (Figs. 1b and S2c). The resulting Cu_SA_-X/MoS_2_ precursors (Fig. S3) were subjected to galvanic replacement in K_2_PtCl_4_ under open-circuit conditions, yielding atomically dispersed Pt on ce-MoS_2_. The gradual change in chronopotentiometry potential from 0.15 to 0.45 V over 600 s confirms complete substitution of Cu by Pt (Fig. S2d). The corresponding catalysts are designated as Pt_SA_-0.1/MoS_2_, Pt_SA_-0.14/MoS_2_, Pt_SA_-0.18/MoS_2_, Pt_SA_-0.24/MoS_2_, and Pt_SA_-0.3/MoS_2_.

The HR-TEM images of Pt_SA_-0.1/MoS_2_ (Fig. S4) show that the ce-MoS_2_ morphology is retained, with a lattice spacing of 0.64 nm corresponding to the (002) plane of MoS_2_ [33]. No obvious Pt-containing clusters or nanoparticles are observed in either TEM or HR-TEM images. HAADF-STEM image of Pt_SA_-0.1/MoS_2_ reveals both 2H- and 1T-phase domains [34], consistent with Raman analysis (Fig.S4c-f). The decreased intensity of the E^1^_2g_ and A_1g_ modes in Pt_SA_-0.1/MoS_2_ relative to ce-MoS_2_ indicates a spontaneous 2H → 1 T phase transition upon Cu/Pt decoration [35]. Brighter atomic columns in HAADF-STEM images, attributable to the higher atomic number of Pt relative to Mo and S [36], confirming atomically dispersed Pt on the ce-MoS_2_ nanosheets (encircled by red circles in Fig. 1c). The energy-dispersive X-ray spectroscopy (EDS) elemental mapping of Pt_SA_-0.1/MoS_2_ further shows uniform distribution of Pt atoms without distinct aggregation (Fig. 1d). Atomic-scale HAADF-STEM images of all Pt_SA_-X/MoS_2_ samples (X = 0.1–0.3) reveal exclusively single-atom Pt sites (Figs. S5-S10). Intensity profile analysis in Fig. S4a, b shows that the neighboring Pt···Pt distances (0.297 nm) exceed that in metallic Pt (0.258–0.279 nm) [37], further confirming the isolated single-atom nature and absence of atomic clusters.

Importantly, the density of bright Pt atomic features increases systematically with decreasing deposition potential, evidencing tunable enrichment of Pt_adj_ sites via the UPD strategy. HAADF-STEM analysis reveals that Pt_SA_-0.3/MoS_2_ dominated by Pt_iso_ sites (blue circles in Fig. 1e), whereas Pt_SA_-0.1/MoS_2_ by Pt_adj_ sites (pink rectangles, Fig. 1g), and intermediate samples contain both configurations (Fig. 1f). The results demonstrate that potential-controlled deposition enables precise atomic-scale engineering of non-bonded Pt_adj_ sites. ICP-OES measurements provide the mass loading of Pt for Pt_SA_-0.1/MoS_2_, Pt_SA_-0.14/MoS_2_, Pt_SA_-0.18/MoS_2_, Pt_SA_-0.24/MoS_2,_ and Pt_SA_-0.3/MoS_2_ is 5.4%, 4.0%, 2.3%, 1.2%, and 0.96%, respectively. Combined HAADF-STEM and ICP-OES analyses reveal a direct correlation between Pt content and the enrichment of adjacent single-atom Pt sites.

**Fig. 2:**
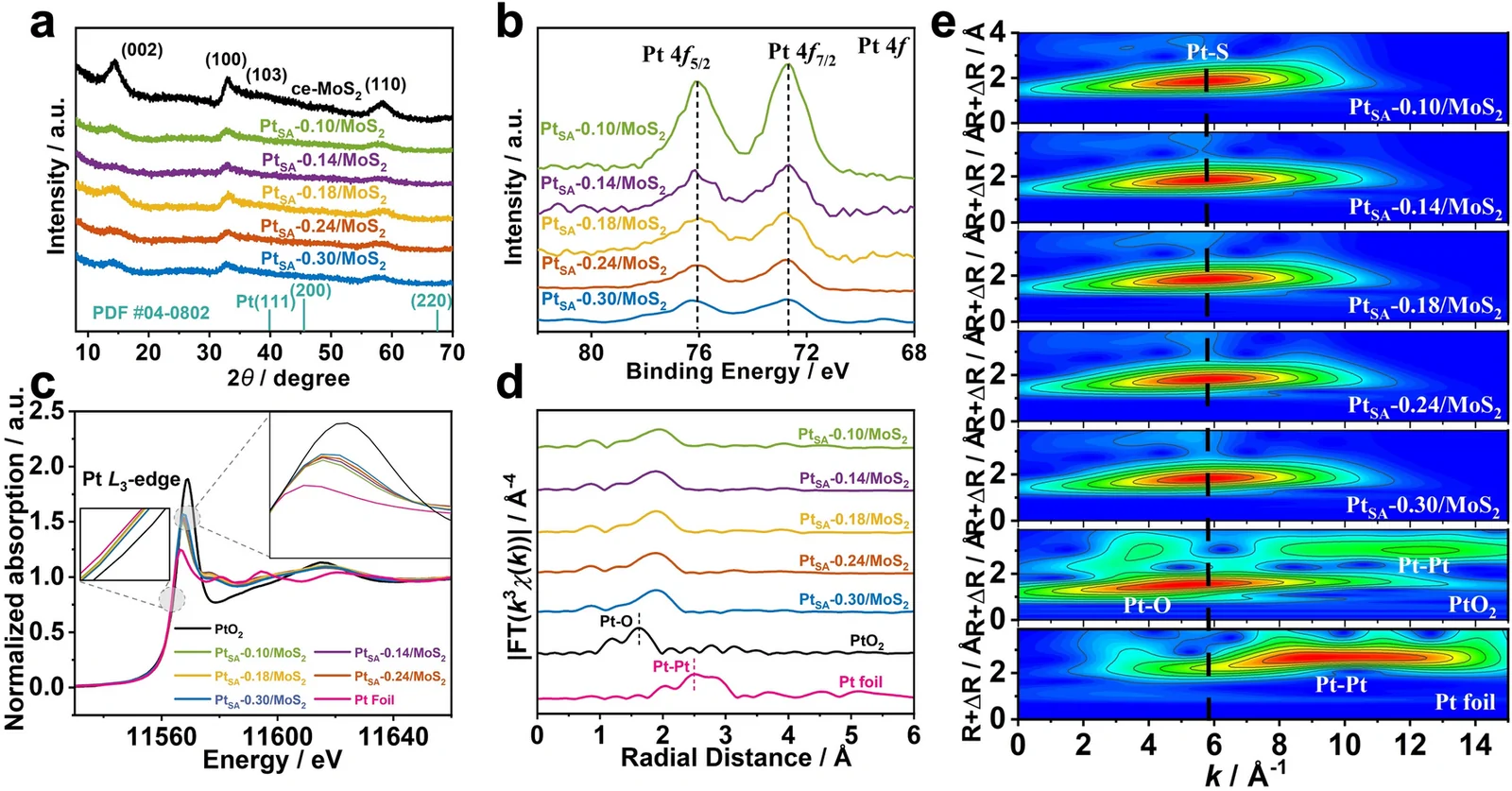
Electronic properties of Pt_SA_-X/MoS_2_. **a** XRD patterns of the Pt_SA_-X/MoS_2_ and pure ce-MoS_2_ samples. **b** Pt 4*f* XPS spectra of Pt_SA_-X/MoS_2_. **c** Normalized XANES spectra at the Pt *L*_3_-edge of Pt_SA_-X/MoS_2_, Pt foil and PtO_2_. **d** Corresponding *k*^3^-weighted Fourier Transform spectra and **e** Wavelet Transform spectra of Pt_SA_-X/MoS_2_, Pt foil and PtO_2_

The XRD patterns of Pt_SA_-X/MoS_2_ (Fig. 2a) closely resemble those of the pristine ce-MoS_2_ sample, with no detectable diffraction peaks relating to Pt nanostructures [28], consistent with TEM and HAADF-STEM observations. The XPS was employed to investigate the surface composition and electronic states of ce-MoS_2_, Pt_SA_-X/MoS_2_, and commercial Pt/C catalysts. High-resolution XPS (HR-XPS) of Mo 3*d* and S 2*p* in Pt_SA_-0.1/MoS_2_ exhibit a negative shift compared to pristine ce-MoS_2_ (Fig. S11a, b), suggesting electron gain by Mo and S upon incorporation of single atomic Pt [28, 38]. This shift is attributed to the strong EMSI between Pt and the ce-MoS_2_, facilitating electron transfer from single-atom Pt to ce-MoS_2_ support [39]. The Pt 4*f* spectrum of Pt_SA_-0.1/MoS_2_ shows a significant positive shift compared with commercial Pt/C (Fig.S11c), reflecting a markedly altered electronic state of single-atom Pt due to differences in oxidation state. HR-XPS analysis of Pt 4*f*, Mo 3*d*, S 2*p*, and Cu 2*p* regions for Cu-deposited intermediate and Pt_SA_-X/MoS_2_ confirms the absence of Cu 2*p* signals (Figs. S3d and S12a), evidencing complete galvanic replacement of Cu adatoms by Pt. While Mo 3*d* and S 2*p* peak positions remain largely unchanged with decreasing deposition potential (Fig.S12b, c), the Pt 4*f* peaks exhibit a systematic negative shift (Fig. 2b), demonstrating that the electronic states of single-atom Pt are precisely tunable [33].

XAFS analyses, including both X-ray absorption near-edge structure (XANES) and EXAFS, were conducted to further investigate the detailed electronic nature and coordination environments of Pt atoms in different Pt_SA_-X/MoS_2_ samples. The normalized Pt *L*_3_-edge XANES spectra lie between those of PtO_2_ (+ 4) and Pt foil (0) (Fig. 2c), indicating presence of oxidized Pt species [40]. The white-line intensity decreases from Pt_SA_-0.3/MoS_2_ to Pt_SA_-0.1/MoS_2_, confirming a relatively lower valence state of Pt in Pt_SA_-0.1/MoS_2_. Fourier-transformed (FT) *k*^3^-weighted EXAFS spectra (Fig. 2d) display a dominant peak at 1.93 Å, assigned to the Pt–S first-shell contribution [41]. The EXAFS spectra fit well with the simulated Pt–S_4_/MoS_2_ model (Figs. S13-S18 and Table S1) [42–44]. The wavelet transform (WT) analysis of the *k*^3^-weighted EXAFS spectra shows a single intensity maximum at approximately 1.9 Å in the R space and 5.9 Å^−1^ in the *k* space (Fig. 2e), distinguishing it from Pt foil [45]. Collectively, these results confirm the controlled modulation of Pt oxidation states and the enrichment of Pt_adj_ sites within the 2D MoS_2_ matrix.

### Electrocatalytic HER activity on Pt_adj_***vs*** Pt_iso_

**Fig. 3:**
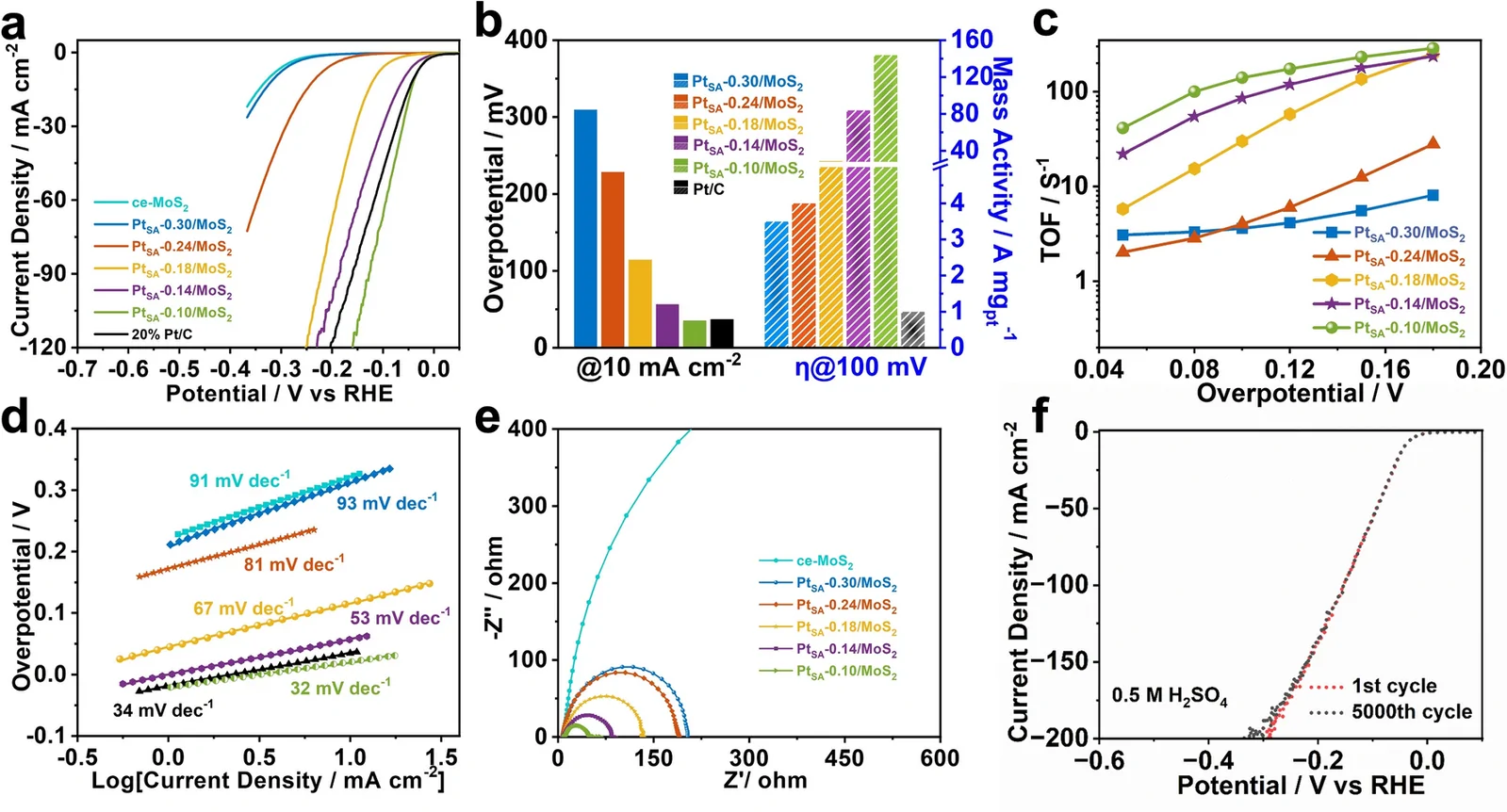
Electrocatalytic HER performance of Pt_SA_-X/MoS_2_. **a** HER polarization curves of pure ce-MoS_2_, Pt_SA_-X/MoS_2_, and 20% commercial Pt/C samples with the same loading on the working electrode in Ar-saturated solution of 0.5 M H_2_SO_4_ at a scan rate of 20 mV s^−1^. **b** Corresponding overpotentials at 10 mA cm^−2^ and mass activity normalized to the Pt loading at an overpotential of 100 mV of Pt_SA_-X/MoS_2_ and 20% commercial Pt/C samples in a solution of 0.5 M H_2_SO_4_. **c** Corresponding TOF values and **d** Tafel slopes of Pt_SA_-X/MoS_2_ and 20% commercial Pt/C samples in a solution of 0.5 M H_2_SO_4_. **e** Electrochemical impedance spectra of Pt_SA_-X/MoS_2_ and ce-MoS_2_ at the onset potentials (obtained at the current density was 0.5 mA cm^−2^) of each electrocatalysts in a solution of Ar-saturated solution of 0.5 M H_2_SO_4_. **f** Stability test of Pt_SA_-0.1/MoS_2_ by potential cycling before and after 5000 cycles

LSV results in acidic electrolyte demonstrate that the Pt_SA_-X/MoS_2_ samples significantly enhance the HER activity and reduce the onset potential compared to the pristine MoS_2_, particularly for Pt_SA_-0.1/MoS_2_ (Fig. 3a). The Pt_SA_-0.1/MoS_2_ reaches a standard reference current density of 10 mA cm⁻^2^ at an overpotential of only 35 mV, outperforming Pt_SA_-0.14/MoS_2_ (57 mV), Pt_SA_-0.18/MoS_2_ (115 mV), Pt_SA_-0.24/MoS_2_ (229 mV), Pt_SA_-0.3/MoS_2_ (310 mV), and even commercial 20% Pt/C (38 mV). Selective blocking of Pt sites by SCN⁻ (from potassium thiocyanate), via formation of Pt–SCN covalent bonds, dramatically suppresses HER activity in Pt_SA_-X/MoS_2_ (Fig. S19), confirming that catalysis originates from single-atom Pt sites [46]. The normalized mass activity of Pt_SA_-0.1/MoS_2_ at an overpotential of 100 mV is 144 A mg_Pt_^−1^, exceeding the commercial Pt/C and Pt_SA_-0.3/MoS_2_ by factors of 144 and 41-fold, respectively (Fig. 3b). This pronounced enhancement highlights that increased enrichment of Pt_adj_ sites in Pt_SA_-0.1/MoS_2_ provides a decisive advantage in further boosting HER activity. Additionally, the calculated turnover frequency (TOF) (Fig. 3c) values for Pt_SA_-0.1/MoS_2_, Pt_SA_-0.14/MoS_2_, Pt_SA_-0.18/MoS_2_, Pt_SA_-0.24/MoS_2_, and Pt_SA_-0.3/MoS_2_ are 43.1, 22.5, 5.9, 3.2, and 2.0 s^−1^ at the overpotential of 50 mV, respectively, indicating that Pt_SA_-0.1/MoS_2_ exhibits the highest intrinsic electrocatalytic activity for HER [47]. The outstanding HER performance of Pt_SA_-0.1/MoS_2_ is further corroborated by Tafel plots, where Tafel slope (Fig. 3d) for Pt_SA_-0.1/MoS_2_ is 32 mV dec^−1^, smaller than those of Pt_SA_-0.14/MoS_2_ (53 mV dec^−1^), Pt_SA_-0.18/MoS_2_ (67 mV dec^−1^), Pt_SA_-0.24/MoS_2_ (81 mV dec^−1^), Pt_SA_-0.3/MoS_2_ (93 mV dec^−1^), MoS_2_ (91 mV dec^−1^), and Pt/C (34 mV dec^−1^), indicating that the accelerated HER kinetics observed at Pt_SA_-0.1/MoS_2_ are consistent with a Volmer–Tafel (VT) mechanism [48]. In contrast, the Pt_SA_-0.3/MoS_2_ catalyst with purely isolated Pt active sites, exhibits the Volmer–Heyrovsky (VH) route. EIS results show that Pt_SA_-0.1/MoS_2_ has the lowest charge- transfer resistance (*R*_ct_) in the acidic medium among all the Pt_SA_-X/MoS_2_ samples and the pristine MoS_2_, confirming its superior charge-transfer properties in HER (Fig. 3e), consistent with the LSV and Tafel analysis. The Pt_SA_-0.1/MoS_2_ catalyst demonstrates remarkable long-term durability in 0.5 M H_2_SO_4_ electrolyte (Figs. 3f and S20). Overall, the electrocatalytic HER performance of Pt_SA_-0.1/MoS_2_ is superior or comparable to previously reported catalysts (Fig. S21 and Table S2).

Structural and morphological analyses (Figs. 1 and 2) reveal that decreasing the deposition potential from 0.3 to 0.1 V lowers the average Pt oxidation state from 2.40 to 1.97, while simultaneously increasing the enrichment of Pt_adj_ sites (Fig. S22 and TableS3). The notable enhancement in HER activity could thus arise from changes in oxidation state, enriched Pt_adj_ sites, or a combination of both factors. To decouple these effects, we examined Pt SACs on three additional 2D TMD supports (WS_2_, MoSe_2_, WSe_2_). Variations in chalcogen element (S and Se) alter the UPD potential (Fig. S23). HAADF-STEM confirms atomically dispersed Pt on all Pt_SA_-X/TMDs (Pt_SA_-0.1/MoS_2_, Pt_SA_-0.1/WS_2_, Pt_SA_-0/MoSe_2_, Pt_SA_-0/WSe_2_) with similar single-atom arrangements (Fig. S25a). The average Pt oxidation state varies widely, from 1.21 to 2.63 (Fig. S26 and Table S3), consistent with previous reports [23]. Despite these differences, all Pt_SA_-X/TMDs exhibited comparable HER activity (Fig. S25b), indicating that oxidation state is not the dominant factor. We therefore conclude that the enrichment of adjacent Pt sites is the primary determinant of HER performance, as we already observed in Fig. 3b. This prominent promoting effect of Pt_adj_ is also observed in the case of Pt_SA_-X/WS_2_ and Pt_SA_-X/WSe_2_, which follows the same activity trend as Pt_SA_-X/MoS_2_ (Figs. S27 and S28).

### Understanding the Stabilization of Reaction Intermediates on Pt_adj_ Versus Pt_iso_ Sites

**Fig. 4:**
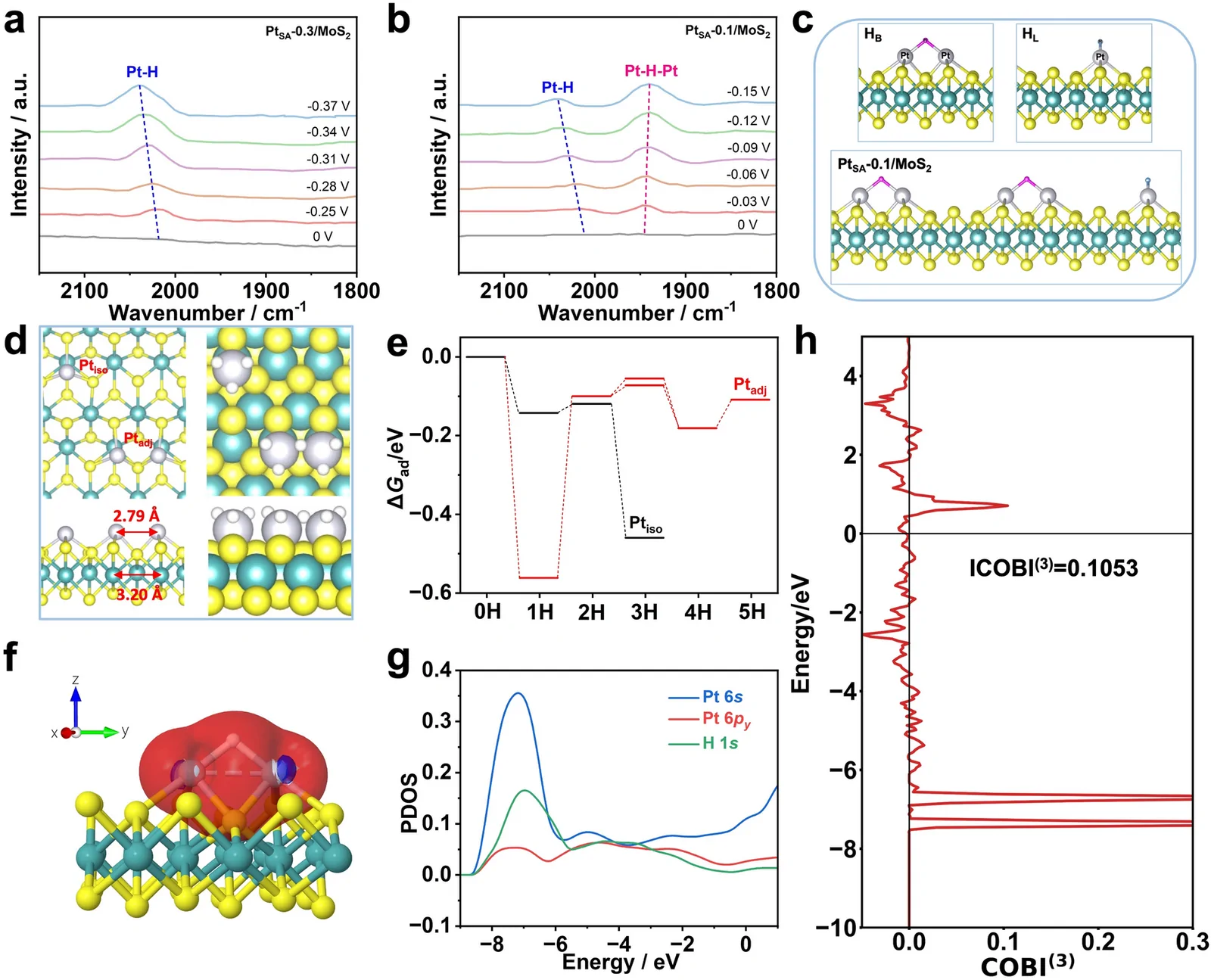
*In situ* ATR-FTIR and theoretical investigations. IR spectra of hydrogen intermediate (*H) on **a** Pt_SA_-0.3/MoS_2_ (Pt_iso_) and **b** Pt_SA_-0.1/MoS_2_ (Pt_adj_). **c** Schematic illustration of hydrogen adsorption configurations on the catalytic surface of Pt_SA_-0.1/MoS_2_. **d** Top and side views of models used for DFT calculations. Left panel shows the structures of Pt_iso_ and Pt_adj_ on MoS_2_ and right panel shows the corresponding Pt atoms with full H adsorption. The adjacent single Pt atom sites are close to each other compared to the Mo site. **e** Hydrogen adsorption free energies on Pt_iso_ and Pt_adj_ in vacuum. **f** Projected wave function of Pt and H atoms. The model is constructed based on the coordinate axes in the left. **g** 6*s* and 6*p*_*y*_ PDOS of adjacent Pt and the 1*s* PDOS of bridging hydrogen. **h** Three-center COBI plot and ICOBI value for Pt–H–Pt

To probe the stabilization mechanisms of reaction intermediates on Pt_adj_ versus Pt_iso_ sites, we combined ATR-FTIR spectroscopy with DFT calculations, uncovering the origin of the enhanced HER activity at Pt_adj_ sites. In situ ATR-FTIR spectroscopy (1800–2150 cm^−1^) reveals distinct hydrogen adsorption geometries on Pt_iso_ sites (Pt_SA_-0.3/MoS_2_) versus Pt_adj_ sites (Pt_SA_-0.1/MoS_2_) during HER (Fig. 4a, b). The Pt_iso_ sites exhibit a Pt–H stretching band at 2041–2020 cm^−1^, assigned to linear adsorbed hydrogen intermediate (^*^H_L_) bound to single Pt atoms (Fig. 4c), which blue shifts with potential from 0 to -0.37 V *vs*. RHE [49, 50]. In contrast, the Pt_adj_ sites display a dominant 1951–1946 cm^−1^ band, characteristics of bridge hydrogen intermediate (^*^H_B_) coordinated to two Pt atoms (Pt–H–Pt, Fig. 4c) [49]. The ^*^H_B_ band emerges at a significantly lower onset potential than that of ^*^H_L_, confirming thermodynamically more favorable adsorption on Pt_adj_ ensembles. With increasing negative potential, this band intensifies and undergoes a red-shift, indicating enhanced stabilization and dominance. The underlying Pt–H–Pt geometry promotes electron delocalization, weakens the Pt–H bond, and consequently reduces the vibrational frequency. Although a minor feature around 2020 cm^−1^ indicates remain of trace ^*^H_L_, the prevalence of the ^*^H_B_ band highlights a cooperative ensemble effect wherein adjacent Pt atoms collectively stabilize a key hydrogen intermediate [51]. Such stabilization is anticipated to facilitate the Tafel step by pre-organizing hydrogen atoms in a configuration optimal for H–H bond formation, thereby lowering the kinetic barrier.

DFT calculations classify Pt atoms as Pt_iso_ or Pt_adj_ based on local atomic environment (Fig. 4d and structures I, II in Fig. S29), consistent with STEM and XAFS analyses showing that Pt_adj_ proportion increases with Pt loading. Calculated differential free energy changes of multiple hydrogen atom adsorption are presented in Fig. 4e, which reveals surprisingly different trends of evolution for the two types of single Pt atoms. At the Pt_iso_ sites, the first two adsorbed H atoms exhibit similar free energy changes of approximately -0.1 eV, while the third adsorbed hydrogen shows a remarkably strong stabilization, with a drop in free energy change by approximately −0.3 eV. In contrast, at the Pt_adj_ sites, the first deposited hydrogen atom prefers to occupy the bridge position between the Pt atom pair (site c as shown in Fig. S34), which is 0.4–0.5 eV more negative than the subsequently adsorbed hydrogen atoms. These discrepancies could be key to understanding the significant variations in HER performance on Pt_SA_ catalysts fabricated under different deposition potentials. The intensive stabilization of the third hydrogen atom at Pt_iso_ suggests the occurrence of multi-hydrogen underpotential deposition, potentially leading to poisoning of the active sites due to over-binding hydrogen atoms [52]. Desorption of the hydrogen can only proceed via the Heyrovsky mechanism when a large potential bias is applied. However, at the Pt_adj_ site, subsequent hydrogen adsorption shows efficient suppression of adsorption free energy fluctuations, remaining in the optimal region for a typical Volmer–Tafel process [52]. These theoretical assessments are consistent with our experimental observations. The catalyst dominated by Pt_iso_ sites, which is fabricated with a depositing potential of + 0.3 V, exhibits HER performance comparable to the intrinsic activity of MoS_2_ (Fig. 3a). The overpotential is notably greater than that of catalysts prepared at lower deposition potentials, and it is accompanied by a typical Tafel slope indicative of the VH mechanism. Conversely, the experimentally determined Tafel slope of HER at Pt_SA_-0.1/MoS_2_, coupled with the considerably reduced overpotential, strongly suggests that the HER at the Pt_adj_ site should proceed via the VT mechanism.

The Pt–Pt distance in the Pt_adj_ sites is calculated to be 2.8 Å, which is significantly longer than those reported in the previously reported dual-atom Pt catalysts [53–55]. At this adjacent distance, the attraction between the atom pair is still expected, given its discrepancy with the Mo–Mo separation of 3.2 Å in the substrate (Fig. 4d). This structural uniqueness results in a novel chemical bonding between the first adsorbed hydrogen atom and the Pt atom pair, characterized as a “Pt–H–Pt” three-center two-electron structure, as shown in Fig. 4f. Surprisingly, the 6*p* atomic orbital of the Pt atoms plays a crucial role in stabilizing the bridging hydrogen, as revealed by the highly overlapping partial density of states (PDOS) of Pt 6*s*, Pt 6*p*_*y*_*,* and H 1*s* orbitals in the Pt–H–Pt moiety, as shown in Fig. 4g. Using the crystal orbital bond index (COBI) analysis method [56], it is found that the participation of the 6*p* atomic orbital of the Pt atoms occurs through the formation of *sp* hybrid orbitals, which combine with the 1*s* orbital of the bridging hydrogen atom to form the three-center bond structure. The COBI function is illustrated in Fig. 4h. Integration of the COBI function up to the Fermi level provides an ICOBI index of 0.1053, indicating a moderately strong three-center chemical bond. Due to its involvement in this novel three-center chemical bond, the bridging hydrogen is negatively charged, contrasting with all other positively charged hydrogen atoms. It is reasonable to propose that the property of the adjacently deposited Pt atom pair can also be significantly modulated by the bridging hydrogen, enhancing their performance in catalyzing the HER process.

In essence, the potential of zero charge (PZC) plays an important role in shaping the HER mechanism. In principle, a fully explicit description of the electrolyte combined with sufficient sampling of phase space would yield the most accurate description of the interfacial fields and PZC values, as demonstrated by Cheng and co-workers [57]. However, the prohibitive computational cost of *ab initio* molecular dynamics (AIMD) makes it impractical for our model systems. Alternatively, the implicit solvation model treats the solvent as a continuous medium, which can significantly reduce computational costs by avoiding the expensive calculation of explicit water molecules. Although it may introduce systematic deviations in the computed absolute PZC values (*e.g.*, the absolute PZC is overestimated by approximately 0.5 V for the Pt (111) electrode [58]), it is still useful for assessing the electron density redistribution in systems of interest, as noted earlier by Nørskov, Head-Gordon, and co-workers [59]. Moreover, this study does not aim to quantitatively evaluate the PZC, but rather to reveal the qualitative trend of PZC variation with the Pt loading and conformation of atom deposition, which is expected to be less affected by the solvent effect treatment, as similar electrode/electrolyte structures should cancel out a significant portion of the errors. Accordingly, a linear Poisson–Boltzmann equation-based implicit solvation model implemented in the VASPsol code [58] was employed to evaluate the differences in PZCs in a few models of Pt deposition on mono-layer MoS_2_.

To inspect the impact of Pt loading on PZC, two models named as III and IV are created in addition to the structures I and II (Fig. S29). Obviously, the calculated PZC for all four structures listed in Table S4 presents consistent negative shift with increasing Pt loading. More positive PZC implies that more negative electric charge will be injected into the electrode at the same operating potential (Table S5), leading to a stronger electrode/electrolyte interfacial electric field. Accordingly, on electrodes with low Pt loading dominated by Pt_iso_, a stronger electric field will impede the transport of hydronium or hydroxyl ions within the electric double layer region by rigidifying the interfacial water molecules, which will increase the reaction overpotential [60, 61], as well as pushing the reaction mechanism to the slower Volmer–Heyrovsky pathway. Conversely, on higher Pt loading electrodes, the much weaker interfacial electric field due to more negative PZC will facilitate the Volmer–Tafel mechanism because the looser water network at the interface does not hinder the approaching of two leaving hydrogen atoms.

## Conclusions

In summary, a site-specific UPD-galvanic displacement strategy enables precise tuning of non-bonded adjacent-site proximity and Pt oxidation state in Pt_SA_-X/MoS_2_. Enriched Pt_adj_ sites, rather than oxidation state, proved as the dominant activity descriptor, delivering a 41-fold higher mass activity than Pt_iso_ sites by stabilizing bridge-H intermediate and lowering the H–H coupling barrier. By establishing adjacent-site proximity as a decisive activity descriptor, this work provides a mechanistic foundation for designing single-atom catalysts with optimized intermediate binding and accelerated reaction kinetics. Although preliminary experimental evidence suggests that similar site-proximity effects may extend to other transition metal dichalcogenide supports in acidic HER, broader generalization across different metal centers and reactions (*e.g.,* CO_2_RR, NRR) will require further validation.

## Supplementary Information

Below is the link to the electronic supplementary material.

Supplementary file1 (DOCX 34606 KB)
