# Supplementary material for: Adjacent-Site Proximity as a Dominant Activity Descriptor in Single-Atom Pt Catalysts for Hydrogen Evolution Reaction
Source: Nanomicro Lett. 2026 May 4;18:350. doi: 10.1007/s40820-026-02201-z (PMC13136461; doi:10.1007/s40820-026-02201-z)
Supplement: Supplementary file 1 — Supplementary file1 (DOCX 34606 KB) [file 40820_2026_2201_MOESM1_ESM.docx]

Supporting Information for

**Adjacent-Site Proximity as a Dominant Activity Descriptor in Single-Atom Pt Catalysts for Hydrogen Evolution Reaction**

Xue-Lu Chen^1^†, Yu-Yang Liu^2^†, Sudip Biswas^1^, Yi Yang^1^, Yi Shi^3^*, Chun-Gen Liu^2^*, Xing-Hua Xia^1^*

^1^State Key Laboratory of Analytical Chemistry for Life Science, School of Chemistry and Chemical Engineering, Nanjing University, Nanjing 210023, P. R. China

^2^Institute of Theoretical and Computational Chemistry, School of Chemistry and Chemical Engineering, Nanjing University, Nanjing 210023, P. R. China

^3^School of Chemistry and Molecular Engineering, East China Normal University, Dongchuan Road 500, Shanghai 200241, P. R. China

†Xue-Lu Chen and Yu-Yang Liu contributed equally to this work.

*Corresponding authors. Email: [yshi@chem.ecnu.edu.cn](mailto:yshi@chem.ecnu.edu.cn) (Yi Shi); [cgliu@nju.edu.cn](mailto:cgliu@nju.edu.cn) (Chun-Gen Liu); [xhxia@nju.edu.cn](mailto:xhxia@nju.edu.cn) (Xing-Hua Xia)

**S1 Supplementary Calculation Notes**

**Note S1.1 Calculation of Mass Activity for HER (𝜂@100 mV)**

Pt_SA_-0.1/MoS_2_:

$$\begin{aligned} MA=\frac{67.45\times0.07065\times{10}^{-3}}{6.25\times{10}^{-4}\times5.4 \%}=141.20 A {mg}^{-1}\# \end{aligned}$$

Pt_SA_-0.14/MoS_2_:

$$\begin{aligned} MA=\frac{29.90\times0.07065\times{10}^{-3}}{6.25\times{10}^{-4}\times4.0 \%}=84.50 A {mg}^{-1}\# \end{aligned}$$

Pt_SA_-0.18/MoS_2_:

$$\begin{aligned} MA=\frac{6.06\times0.07065\times{10}^{-3}}{6.25\times{10}^{-4}\times2.3 \%}=29.77 A {mg}^{-1}\# \end{aligned}$$

Pt_SA_-0.24/MoS_2_:

$$\begin{aligned} MA=\frac{0.43\times0.07065\times{10}^{-3}}{6.25\times{10}^{-4}\times1.2 \%}=4.05 A {mg}^{-1}\# \end{aligned}$$

Pt_SA_-0.3/MoS_2_:

$$\begin{aligned} MA=\frac{0.3\times0.07065\times{10}^{-3}}{6.25\times{10}^{-4}\times0.96 \%}=3.53 A {mg}^{-1}\# \end{aligned}$$

20% Pt/C:

$$MA=\frac{48.03\times0.07065\times{10}^{-3}}{2\times8\times{10}^{-3}\times20 \%}=1.06 A {mg}^{-1}$$

**Note S1.2 Calculation of TOFs for HER**

Pt_SA_-0.1/MoS_2_:

$$TOF(@0.05 V)=\frac{I}{2F\times n}=\frac{19.59\times0.07065\times{10}^{-3}}{2\times96500\times\frac{6.25\times{10}^{-4}\times5.4 \%\times{10}^{-3}}{195}}=41.45 S^{-1}$$

$$TOF(@0.10 V)=\frac{67.45\times0.07065\times{10}^{-3}}{2\times96500\times\frac{6.25\times{10}^{-4}\times5.4 \%\times{10}^{-3}}{195}}=140.62 S^{-1}$$

$$TOF(@0.15 V)=\frac{109.88\times0.07065\times{10}^{-3}}{2\times96500\times\frac{6.25\times{10}^{-4}\times5.4 \%\times{10}^{-3}}{195}}=232.50 S^{-1}$$

Pt_SA_-0.14/MoS_2_:

$$TOF(@0.05 V)=\frac{7.69\times0.07065\times{10}^{-3}}{2\times96500\times\frac{6.25\times{10}^{-4}\times4.0 \%\times{10}^{-3}}{195}}=22.00 S^{-1}$$

$$TOF(@0.10 V)=\frac{29.90\times0.07065\times{10}^{-3}}{2\times96500\times\frac{6.25\times{10}^{-4}\times4.0 \%\times{10}^{-3}}{195}}=85.49 S^{-1}$$

$$TOF(@0.15 V)=\frac{62.65\times0.07065\times{10}^{-3}}{2\times96500\times\frac{6.25\times{10}^{-4}\times4.0 \%\times{10}^{-3}}{195}}=179.16 S^{-1}$$

Pt_SA_-0.18/MoS_2_:

$$TOF(@0.05 V)=\frac{1.17\times0.07065\times{10}^{-3}}{2\times96500\times\frac{6.25\times{10}^{-4}\times2.3 \%\times{10}^{-3}}{195}}=5.79 S^{-1}$$

$$TOF(@0.10 V)=\frac{6.06\times0.07065\times{10}^{-3}}{2\times96500\times\frac{6.25\times{10}^{-4}\times2.3 \%\times{10}^{-3}}{195}}=30.10 S^{-1}$$

$$TOF(@0.15 V)=\frac{27.32\times0.07065\times{10}^{-3}}{2\times96500\times\frac{6.25\times{10}^{-4}\times2.3 \%\times{10}^{-3}}{195}}=135.69 S^{-1}$$

Pt_SA_-0.24/MoS_2_:

$$TOF(@0.05 V)=\frac{0.21\times0.07065\times{10}^{-3}}{2\times96500\times\frac{6.25\times{10}^{-4}\times1.2 \%\times{10}^{-3}}{195}}=2.02 S^{-1}$$

$$TOF(@0.10 V)=\frac{0.43\times0.07065\times{10}^{-3}}{2\times96500\times\frac{6.25\times{10}^{-4}\times1.2 \%\times{10}^{-3}}{195}}=4.02 S^{-1}$$

$$TOF(@0.15 V)=\frac{1.32\times0.07065\times{10}^{-3}}{2\times96500\times\frac{6.25\times{10}^{-4}\times1.2 \%\times{10}^{-3}}{195}}=12.57 S^{-1}$$

Pt_SA_-0.3/MoS_2_:

$$TOF(@0.05 V)=\frac{0.25\times0.07065\times{10}^{-3}}{2\times96500\times\frac{6.25\times{10}^{-4}\times0.96 \%\times{10}^{-3}}{195}}=3.07 S^{-1}$$

$$TOF(@0.10 V)=\frac{0.3\times0.07065\times{10}^{-3}}{2\times96500\times\frac{6.25\times{10}^{-4}\times0.96 \%\times{10}^{-3}}{195}}=3.61 S^{-1}$$

$$TOF(@0.15 V)=\frac{0.46\times0.07065\times{10}^{-3}}{2\times96500\times\frac{6.25\times{10}^{-4}\times0.96 \%\times{10}^{-3}}{195}}=5.55 S^{-1}$$

**S2 Supplementary DFT Notes**

All density functional theory (DFT) calculations were performed using the VASP 5.4.4 package (*1-3*). The core-valence interaction was described by the projector augmented wave (PAW) (*4*) method with an energy cutoff of 500 eV. The exchange and correlation energies were calculated using the RPBE functional (*5*) with the generalized gradient approximation (GGA) (*6*). The k-point mesh was sampled using a 3×3×1 Monkhorst-Pack grid, while a (3×4) MoS_2_ monolayer structure was adopted for the surface simulation, with a slab length of 20 Å. This MoS_2_ (3×4) slab consisted of 12 Mo atoms and 24 S atoms, with the Mo atoms and bottom S atoms frozen during geometry optimization. The criteria for geometry optimization and self-consistent field convergence were set to 0.05 eV/Å and 10^-6^ eV, respectively. Zero-point energies and entropy contributions at room temperature (298.15 K) were calculated from vibrational degrees of freedom with the substrate fixed. All structure optimizations and energy calculations were carried out under vacuum conditions. The potential of zero charge (PZC) was the only property calculated in a solvation environment using the implicit solvation model VASPsol (*7, 8*). These calculations employed a symmetric slab model applied and a slab length of 50 Å. Atomic charges were computed using the atom-in-molecule (AIM) scheme proposed by Bader (*9, 10*). VASPkit (*11*) was used for pre-processing and post-processing of data. The Crystal orbital Hamilton population (COHP) (*12, 13*) and Crystal orbital Bond Index (COBI) (*14*) analysis were performed using the LOBSTER 3.1.0 package (*15*).

The $\Delta G_{H}$ was adopted to theoretically evaluate the catalytic performance for HER, which was calculated by the following equation:

$\Delta G_{H}=\Delta E+\Delta E_{\mathrm{ZPE}}-T\Delta S$ (S-1)

where, $\Delta E$ is the hydrogen adsorption energy computed by the equation $\Delta E=E\left( *H \right)-E\left( * \right)-0.5E(H_{2})$, where $E(*H)$ and $E(*)$ are the total energies of the model with and without hydrogen adsorbed on the binding site *, respectively; and $E(H_{2})$ is the total energy of the single hydrogen molecule. $\Delta E_{\mathrm{ZPE}}$ is the correction of the zero-point energy, $\Delta S$ represents the difference between in entropies between the adsorbed state and the corresponding freestanding state and $T$ is the absolute temperature (298.15 K in this study).

**Note S2.1 Additional Information on Adsorption of Hydrogen Atom**

Figure S31 illustrates the specific adsorption sites of H atoms on isolated Pt. Table S7 lists the detailed adsorption energy data for H atoms at different adsorption sites. Figure S32 provides a further insight in the cases where more than three H atoms adsorbed on Pt_iso_. When one Pt atom adsorbed with four H atoms, two of them were positioned at the distance of just 0.84 Å, indicating the presence of the physically adsorbed H_2_ molecule. It is worth noting that the adsorption energy of the structure with four adsorbed H atoms was reduced lower (0.0185 eV) when compared to the structure with two adsorbed H atoms. This suggests that the energy barrier for H_2_ desorption is extremely low. Consequently, we disregarded the adsorption of four or more H atoms on Pt_iso_ in this context. Figure S33 and Table S8 reveal the adsorption behavior of H on Pt_iso_, considering the presence or absence of surrounding Pt atoms in Structure IV or Structure I. The results indicate that the adsorption of H on Pt_iso_ still largely unaffected by the immediate environment. Table S9 presents data on the vibrational energy and entropy of the H adsorbed on Pt_iso_ in both Structure I and IV, revealing that the environment surrounding the Pt atom has minimal influence.

Figure S34 depicts the adsorption sites of H on Pt_adj_, while Tables S10-S11 provide calculation results for adsorption energy, along with corresponding vibrational energy and entropy. As shown in Figure S35, there exist two distinct configurations when three H atoms are adsorbed on Pt_adj_. One of these configurations can trigger the Tafel reaction, while the other cannot. The energy difference between these configurations is small, allowing for the mutual conversion. Figure S36 shows the influence of varying environments on H adsorption energy on Pt_adj_. Specifically in Structure IV, there is a Pt_iso_ site situated near the Pt_adj_. The Structure 3H@IV is referred to the case where three H atoms already adsorb on the Pt_iso_ within Structure IV. Data presented in Table S12 highlights certain difference in H adsorption energy between Structure II and IV, while the results for Structure II and 3H@IV are remarkable resemblance. Given that Pt_iso_ exhibits affinity for H adsorption compared to the top H on Pt_adj_, we believe that whenever top-H adsorption occurs on Pt_adj_, the Pt_iso_ is already in a state of saturated H adsorption. Therefore, the Structure 3H@IV is more aligned with physical pictures. The resemblance in H adsorption energy findings on Pt_adj_ between Structure II and 3H@IV further suggests that the adsorption environment exerts a limited influence on H adsorption energy on Pt_adj_.

Figures S37-S39 and Table S13 display the adsorption patterns of H on Pt_iso_ and Pt_adj_ based on a series of substrate materials. The representation of adsorption sites in these systems follows the method which is employed for MoS_2_. Our calculations reveal that the adsorption energy of top-H on Pt_adj_ keep low data on various substrate materials, indicating excellent HER reaction activity, which agrees well with the experimental results.

**Note S2.2 Additional Description of the Pt-H-Pt Three-Center Bonding.**

Figure 4c displays the projected wave function derived from the PROCAR file computed by using VASP and visualized with VASPMO. This image depicts the orbitals of Pt and H atoms at an energy level of -7.35 eV relative to the Fermi energy. All pertinent calculations were anchored to the coordinate system displayed in Figure 4c, where the y-axis was aligned with Pt–H–Pt direction. Notably, the image incorporates the non-bonding 5*d*_x2-y2_ component, thereby influencing the shape of the visualized projected wave function. The “Pt–H–Pt” three-center bond had a ICOBI of 0.1053, which was comparable to that in Te–Ge–Te three-center bond (*1*). Figure S40 illustrates the main orbital components of the Pt-H-Pt three-center bond. Detailed analysis demonstrates that 6*p_y_*-1*s*-6*s* (ICOBI=0.0721) constitutes the primary component of the three-center bond, while 6s-1s-6s (ICOBI=0.0266) also contributes to its formation. It is worth noting that although 5*d_yz_*-1*s*-6*s* (ICOBI=0.0251) of Pt 5*d_yz_* orbital makes a positive contribution to the three-center bond, 5*d_yz_*-1*s*-5*d_yz_* (ICOBI=-0.0388) offsets this contribution, indicating that the 5*d_yz_* orbital is unfavorable for the formation of the three-center bond. Then, we can get the conclusion that the three-center bond is formed by the H 1*s* orbital and two hybrid orbitals consist of Pt 6*s* and 6*p_y_* orbitals.

**Supplementary Figures**

**Fig. S1** (a) Low-magnified TEM image and (b) HR-TEM image of chemically exfoliated MoS_2_ (ce-MoS_2_). (c) Low-magnified TEM image of bulk MoS_2_. (d) AFM image of ultrathin MoS_2_ nanosheets and corresponding (e) height profiles of the regions of interest indicated in the AFM image. (f) Raman spectrum of the MoS_2_ sample

**Fig. S2** (a) A 3D scheme of the electrochemical apparatus (the synthetic procedure was described in the method section). (b) CVs of the chemical exfoliated MoS_2_ in an Ar-saturated 0.1 M H_2_SO_4_ solution containing 2 mM CuSO_4_ (black line) or 0.1 M H_2_SO_4_ solution (red line) at a scan rate of 20 mV s^−1^. Peak a is attributed to Cu UPD, whereas peak b corresponds to the bulk deposition of Cu. (c) Corresponding chronoamperometry curves of Cu_SA_-X/MoS_2_. (d) Potential-response curve of Cu adatoms within Pt precursor at the open-circuit potential


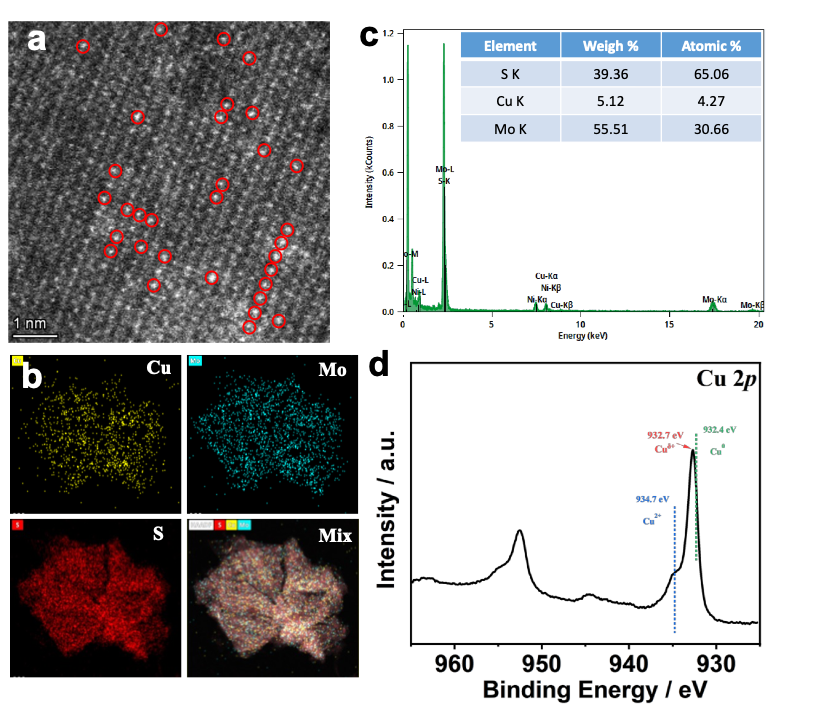


**Fig. S3** (a) HAADF-STEM image of Cu_SA_-0.1/MoS_2_, with red circles highlighting the single atoms of Cu distributed in the MoS_2_. (b) EDS mapping images, (c) EDS spectrum and (d) Cu 2*p* XPS spectrum of Cu_SA_-0.1/MoS_2_ catalyst


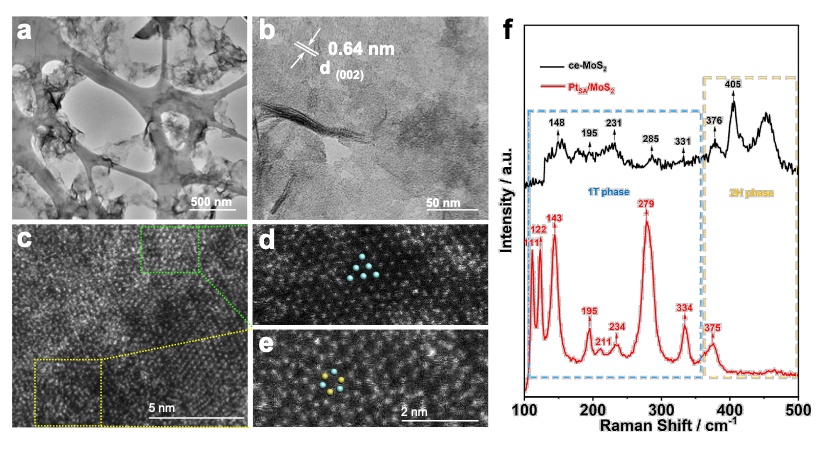


**Fig. S4** (a) Low-magnified TEM and (b) HR-TEM images of Pt_SA_-MoS_2_. (c) HAADF-STEM image of Pt_SA_-MoS_2_. (d) Amplified image of the region enclosed by the green circle of (c) and schematic structure of the unit cells of the 1T-phase MoS_2_. (e) Amplified image of the region enclosed by the yellow rectangle in (c) and schematic structure of the unit cells of the 2H-phase MoS_2_. (f) Raman spectrum of the ce-MoS_2_ sample and Pt_SA_-MoS_2_ sample


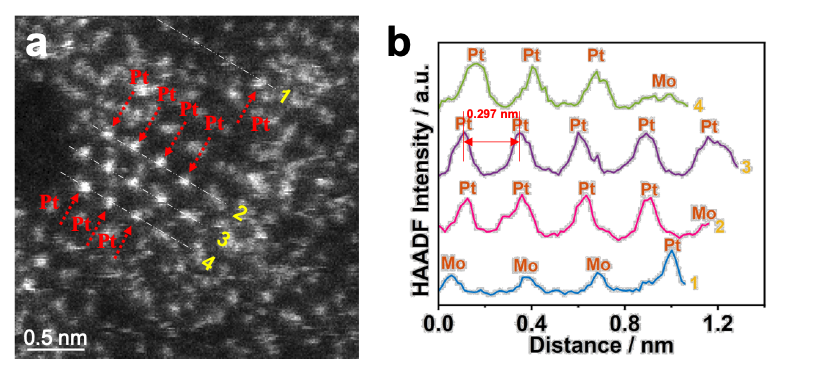


**Fig. S5** (a) HAADF-STEM image and (b) corresponding linear intensity distributions along lines of Pt_SA_-0.1/MoS_2_


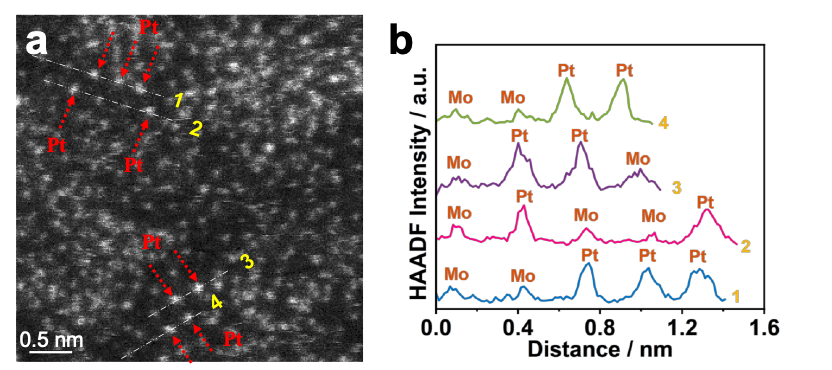


**Fig. S6** (a) HAADF-STEM image and (b) corresponding linear intensity distributions along lines of Pt_SA_-0.14/MoS_2_


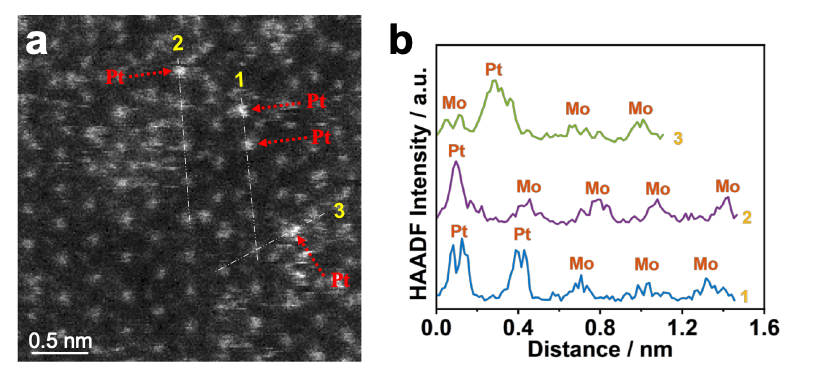


**Fig. S7** (a) HAADF-STEM image and (b) corresponding linear intensity distributions along lines of Pt_SA_-0.18/MoS_2_


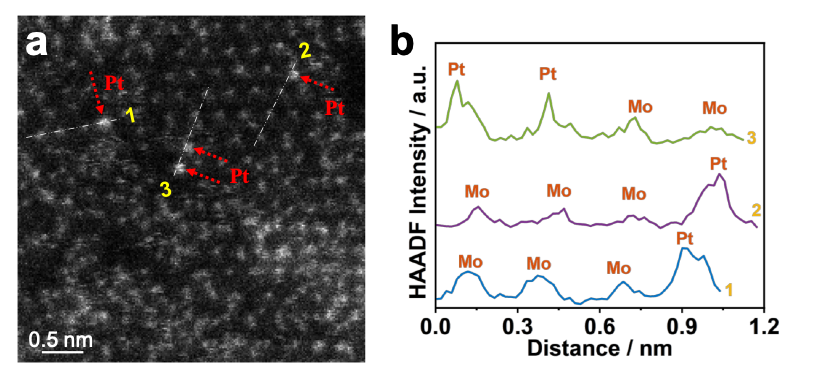


**Fig. S8** (a) HAADF-STEM image and (b) corresponding linear intensity distributions along lines of Pt_SA_-0.24/MoS_2_


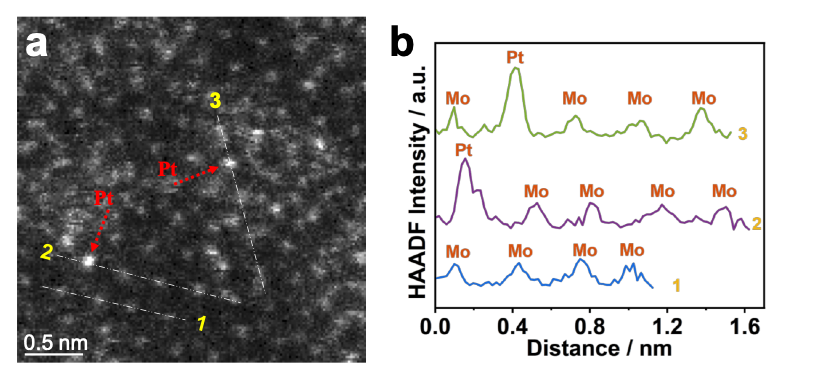


**Fig. S9** (a) HAADF-STEM image and (b) corresponding linear intensity distributions along lines of Pt_SA_-0.3/MoS_2_


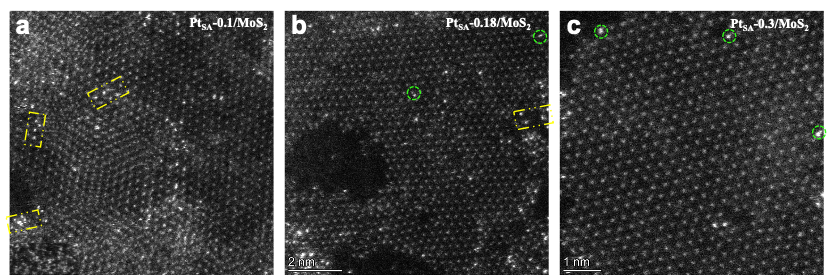


**Fig. S10** HAADF-STEM images of (a) Pt_SA_-0.1/MoS_2_, (b) Pt_SA_-0.18/MoS_2_ and (c) Pt_SA_-0.3/MoS_2_. Inset, Pt_iso_ sites and Pt_adj_ sites are respectively represented in green circle and yellow rectangle

**Fig. S11** (a) Mo 3*d* and (b) S 2*p* XPS spectra of Pt_SA_-0.1/MoS_2_ and ce-MoS_2_. (c) Pt 4*f* XPS spectra of Pt_SA_-0.1/MoS_2_ and commercial Pt/C

**Fig. S12** (a) Cu 2*p,* (b) Mo 3*d* and (c) S 2*p* XPS spectra of Pt_SA_-X/MoS_2_

**Fig. S13** (a) Fitting of the magnitude of the Fourier transform of the *k*^3^-weighted EXAFS (data-blue and fit-red) for Pt_SA_-0.1/MoS_2_ and (b) corresponding EXAFS fitting curve at *k* space

**Fig. S14** (a) Fitting of the magnitude of the Fourier transform of the *k*^3^-weighted EXAFS (data-blue and fit-red) for Pt_SA_-0.14/MoS_2_ and (b) corresponding EXAFS fitting curve at *k* space

**Fig. S15** (a) Fitting of the magnitude of the Fourier transform of the *k*^3^-weighted EXAFS (data-blue and fit-red) for Pt_SA_-0.18/MoS_2_ and (b) corresponding EXAFS fitting curve at *k* space

**Fig. S16** (a) Fitting of the magnitude of the Fourier transform of the *k*^3^-weighted EXAFS (data-blue and fit-red) for Pt_SA_-0.24/MoS_2_ and (b) corresponding EXAFS fitting curve at *k* space

**Fig. S17** (a) Fitting of the magnitude of the Fourier transform of the *k*^3^-weighted EXAFS (data-blue and fit-red) for Pt_SA_-0.30/MoS_2_ and (b) corresponding EXAFS fitting curve at *k* space

**Fig. S18** (a) Fitting of the magnitude of the Fourier transform of the *k*^3^-weighted EXAFS (data-blue and fit-red) for Pt_SA_-X/MoS_2_ and Pt foil and (b) corresponding EXAFS fitting curves of samples and standard at *k* space

**Fig. S19** Current-time curve of (a) Pt_SA_-0.1/MoS_2_, (b) Pt_SA_-0.14/MoS_2_, (c) Pt_SA_-0.18/MoS_2_, (d) Pt_SA_-0.24/MoS_2_, (e) Pt_SA_-0.3/MoS_2_, and (f) ce-MoS_2_ before and after the injection of SCN^-^ ions in Ar-saturated 0.5 M H_2_SO_4_ solution

**Fig. S20** Chronoamperometry test of Pt_SA_-0.1/MoS_2_ at -0.03 V for 20000 s in an Ar-saturated solution of 0.5 M H_2_SO_4_

**Fig. S21** Comparison of HER activity for Pt_SA_-0.1/MoS_2_ in 0.5 M H_2_SO_4_ solution with the state-of-the-art single-atom catalysts reported previously

**Fig. S22** Pt 4*f* HR-XPS spectra of Pt_SA_-X/MoS_2_ samples

**Fig. S23** CVs of the ce-TMDs modified GCE in an Ar-saturated solution of 0.1 M H_2_SO_4_ + 2 mM CuSO_4_. Inset shows the photographs of good-dispersible ce-TMDs nanosheets suspension in water


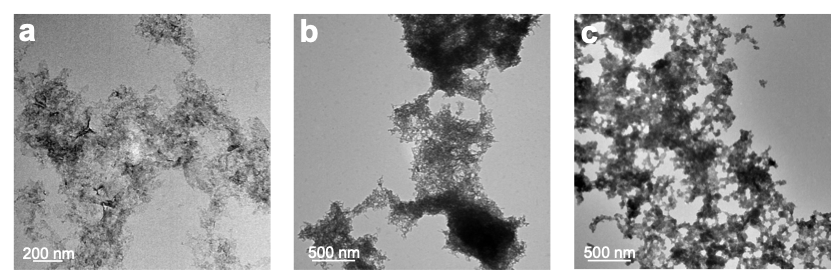


**Fig. S24** Conventional TEM images of the freshly prepared (a) Pt_SA_-WS_2_, (b) Pt_SA_-MoSe_2_ and (c) Pt_SA_-WSe_2_ samples


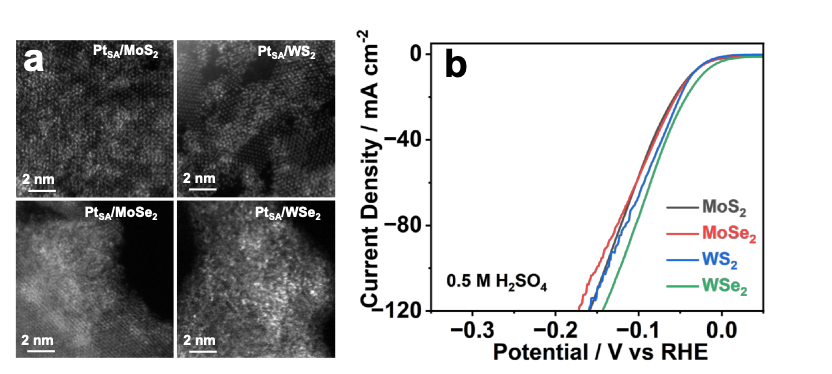


**Fig. S25** (a) HAADF-STEM images of Pt_SA_-MoS_2_, Pt_SA_-WS_2_, Pt_SA_-MoSe_2_, and Pt_SA_-WSe_2_ samples. (b) HER polarization curves of Pt_SA_-MoS_2_, Pt_SA_-WS_2_, Pt_SA_-MoSe_2_, and Pt_SA_-WSe_2_ samples in an Ar-saturated solution of 0.5 M H_2_SO_4_ at a scan rate of 50 mV s^-1^

**Fig. S26** (a-b) Pt 4*f* XPS spectra of Pt_SA_-WS_2_ and Pt_SA_-WSe_2_


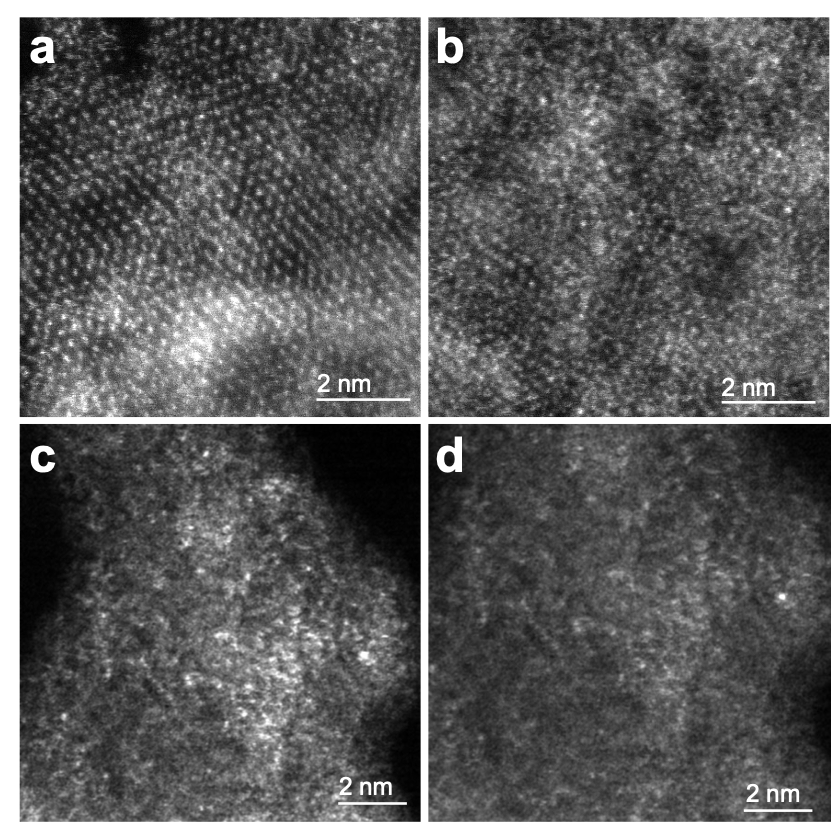


**Fig. S27** HAADF-STEM images of (a) Pt_SA_-0.1/WS_2_, (b) Pt_SA_-0.3/WS_2_, (c) Pt_SA_-0/WSe_2_ and (d) Pt_SA_-0.2/WSe_2_ samples

**Fig. S28** HER polarization curves and mass activity for (a, c) Pt_SA_-X/WS_2_ and (b, d) Pt_SA_-X/WSe_2_ samples in an Ar-saturated solution of 0.5 M H_2_SO_4_ at a scan rate of 20 mV s^-1^

**Fig. S29** Four DFT slab models (a) Pt_iso_ (Structure I), (b) Pt_adj_ (Structure II), (c) two Pt_iso_ (Structure III), and (d) one Pt_iso_ plus one Pt_adj_ (Structure IV) of various Pt loadings on MoS_2_

**Fig. S30** DFT models of different numbers of (a) 3 Pt atoms, and (b) 4 Pt atoms in adjacent Pt atoms. It suggests that the adjacent effect of single atom Pt is universal

**Fig. S31** Hydrogen adsorption sites (a, b and c sites in Table S7) on isolated Pt

**Fig. S32** Four hydrogen atoms adsorption (the “a, 2b, c” structure in Table S7) on isolated Pt

**Fig. S33** Adsorption energy of different H on isolated Pt at different structures

**Fig. S34** Hydrogen adsorption sites (a-e sites in Table S10) on adjacent Pt

**Fig. S35** Two different adsorption modes of 3H adsorption on adjacent Pt. (a) Two top-H adsorbed on two Pt atoms, (b) Two top-H adsorbed on one Pt atom

**Fig. S36** (a) Adsorption energy of different H on adjacent Pt at different structures, and (b) DFT slab model of 3H@IV

**Fig. S37** Hydrogen adsorption energies of the most stable structures on Pt_SA_-MoSe_2_

**Fig. S38** Hydrogen adsorption energies of the most stable structures on Pt_SA_-WS_2_

**Fig. S39** Hydrogen adsorption energies of the most stable structures on Pt_SA_-WSe_2_

**Fig. S40** Orbital-wise and total three-center COBI plots for Pt–H–Pt, corresponding to the structure of Pt_adj_ with a bridge-H adsorption. The corresponding integrated ICOBI (ICOBI) values are given. The plot of 6*p_y_*-1*s*-6*s* contains both 6*p_y_*-1*s*-6*s* and 6*s*-1*s*-6*p_y_* components. 5*d_yz_*-1*s*-6*s* is the same

**Supplementary Tables**

| **Table S1** EXAFS fitting parameters of Pt_SA_-X/MoS_2_ and Pt foil | | | | | | |
| --- | --- | --- | --- | --- | --- | --- |
| Sample | Path | CN^a^ | R(Å)^b^ | σ^2^ (Å^2^)^c^ | ΔE_0_(eV)^d^ | R factor |
| Pt *L*_3_-edge (Ѕ_0_^2^=0.899) | | | | | | |
| Pt foil | Pt-Pt | 12* | 2.764±0.002 | 0.0050 | 8.2 | 0.0031 |
| Pt_SA_-0.10/MoS_2_ | Pt-S | 4.3±0.2 | 2.245±0.011 | 0.0081 | 2.5 | 0.0120 |
| Pt_SA_-0.14/MoS_2_ | Pt-S | 3.9±0.2 | 2.241±0.007 | 0.0063 | 0.9 | 0.0065 |
| Pt_SA_-0.18/MoS_2_ | Pt-S | 4.2±0.2 | 2.234±0.010 | 0.0063 | 0.7 | 0.0091 |
| Pt_SA_-0.24/MoS_2_ | Pt-S | 3.9±0.1 | 2.228±0.006 | 0.0060 | 2.6 | 0.0041 |
| Pt_SA_-0.30/MoS_2_ | Pt-S | 4.4±0.2 | 2.230±0.009 | 0.0062 | 1.8 | 0.0046 |

Note: *^a^CN*, coordination number; *^b^R*, the distance between absorber and backscatter atoms; *^c^σ*^2^, the Debye Waller factor value; *^d^ΔE*_0_, inner potential correction to account for the difference in the inner potential between the sample and the reference compound; *R* factor indicates the goodness of the fit. *S*0^2^ was fixed to 0.899, according to the experimental EXAFS fit of Pt foil by fixing *CN* as the known crystallographic value. * This value was fixed during EXAFS fitting, based on the known structure of Pt. Fitting conditions: *k* range：2.0 - 10.5; *R* range: 1.1-2.5; fitting space: R space; *k*-weight = 3. A reasonable range of EXAFS fitting parameters: 0.800 < *Ѕ*_0_^2^ < 1.000; *CN >* 0; *σ*^2^ > 0 Å^2^; |Δ*E*_0_| < 10 eV; *R* factor < 0.02.

**Table S2** Comparison of HER activity for Pt_SA_-0.1/MoS_2_ in 0.5 M H_2_SO_4_ solution with the state-of-the-art single-atom catalysts reported previously

| Catalyst | Tafel slope  (mV dec^-1^) | Mass activity@ overpotential  (A mg_pt_^-1^) | TOF@ overpotential  (s^-1^) | Reference |
| --- | --- | --- | --- | --- |
| **Pt_SA_-0.1/MoS_2_** | **32** | **144@0.1 V** | **43.1@0.05 V** | **This work** |
| Pt@DG | 53 | 26.05@0.1 V | 26.41@0.1 V | [S2] |
| Pt_1_/OLC | 36 | 7.40@0.038 V | 90@0.2 V | [S3] |
| Pt@PCM | 63.7 | NA | 12@0.2 V | [S4] |
| Pt_1_/hNCNC | 24 | 7.60@0.02 V | 7.67@0.02 V | [S5] |
| Pt_1_/NPC | 28 | 2.86@0.025 V | 100@0.1 V | [S6] |
| Pt-AC/DG-150 | 37.8 | 11.78@0.05 V | 15.7@0.05 V | [S7] |
| Pt-AC/DG-300 | 34.8 | 17.93@0.05 V | 13.4@0.05 V | [S7] |
| PtN_x_/TiO_2_ | 34 | 136@0.1 V | 37.97@0.05 V | [S8] |
| Pt-GDY2 | 46.6 | 23.64@0.1 V | 23.9@0.05 V | [S9] |
| Os/CNS | 41 | NA | 10.55@0.05 V | [S10] |
| ALD100Pt/NGNs | 29 | NA | 2.12@0.05 V | [S11] |
| Pt_1_/NMHCS | 56 | 2.07@0.05 V | 2.09@0.05 V | [S12] |
| Pt_doped_@WC_x_ | 20 | 14.28@0.1 V | 1.04@0.05 V | [S13] |
| Pt SASs/AG | 29.33 | 22.4@0.05 V | 1.63@0.04 V | [S14] |
| ALD50Pt/NGNs | 29 | NA | 10.2@0.05 V | [S11] |
| Pt_1_/NMC | 29 | ~8@0.05 V | 8.08@0.05 V | [S15] |
| Pt_1_/NCP | 28.5 | 6.6@0.05 V | 6.72@0.05 V | [S16] |
| NGA-COF@Pt | 21.8 | 18.2@44 mV | 18.4@44 mV | [S17] |
| Pt_1_/N-C | 14.2 | NA | 22.07@50 mV | [S18] |
| Pt/TiB_x_O_y_ | NA | 37.8@50mV | 33.2@50 mV | [S19] |
| Pt_1_/NNGF | 29.1 | 14.6@50mV | 14.1@50 mV | [S20] |
| Pt- PVP/TNR@GC | 27 | 16.5@50mV | 16.67@50 mV | [S21] |
| PtO_x_/TiO_2_ | 52 | NA | 6.95@50 mV | [S8] |
| Pt_1_/PI@CP | 27.5 | 106.9@0.1 V | 35@0.05 V | [S22] |
| PtN_x_/TiO_2_ | 34 | 136@0.1 V | 37.9@0.05 V | [S23] |
| Pt/MoS_2_-NTA/Ti_3_C_2_ | 35 | 46.5@0.1 V | 47.0@0.1 V | [S24] |
| Pt_SA_@Mo_2_C@NC | 26 | 75.21@0.1 V | 14.4@0.1 V | [S25] |
| Pt/np-Co_0.85_Se | 35 | 1.32@0.1 V | 3.93@0.1 V | [S25] |
| Ti_3_C_2_T_x_-Pt_SA_ | 45 | 23.21@0.1 V | 23.45@0.1 V | [S27] |
| PtCu/WO_3_@CF | 45.9 | 7.3@0.1 V | 10.9@0.1 V | [S28] |
| Pt-SAs/MoSe_2_ | 28 | 34.4@0.1 V | 151@0.2 V | [S29] |
| MAC@Pt 1000 | 24.9 | 1.5@0.1 V | 0.52@40 mV | [S30] |
| Pt-SA_0.056_/Mo-L | 31.1 | 2.06@0.1 V | 1.2@140 mV | [S31] |
| Pt-MoAl_1-x_B | 78.8 | 4@0.1 V | 0.63@0.1 V | [S32] |
| 0.22Pt-HMoS_2_ | 34.83 | ~2@0.1 V | NA | [S33] |

**Table S3** Average oxidation state of Pt calculated from the XPS spectra of the Pt_SA_-X/TMDs catalysts

| Sample | Content (%) | Average oxidation state of Pt |
| --- | --- | --- |
| Pt_SA_-0.10/MoS_2_ | 1.3 (Pt^4+^) | 1.97 |
|  | 95.7 (Pt^2+^) |  |
|  | 3.0 (Pt^0^) |  |
| Pt_SA_-0.14/MoS_2_ | 13.0 (Pt^4+^) | 2.10 |
|  | 78.9 (Pt^2+^) |  |
|  | 8.1 (Pt^0^) |  |
| Pt_SA_-0.18/MoS_2_ | 25.5 (Pt^4+^) | 2.22 |
|  | 67.8 (Pt^2+^) |  |
|  | 6.7 (Pt^0^) |  |
| Pt_SA_-0.24/MoS_2_ | 25.5 (Pt^4+^) | 2.37 |
|  | 67.8 (Pt^2+^) |  |
|  | 6.7 (Pt^0^) |  |
| Pt_SA_-0.3/MoS_2_ | 20 (Pt^4+^) | 2.40 |
|  | 80 (Pt^2+^) |  |
| Pt_SA_-0.1/WS_2_ | 11.5 (Pt^4+^) | 1.21 |
|  | 37.5 (Pt^2+^) |  |
|  | 51 (Pt^0^) |  |
| Pt_SA_-0/WSe_2_ | 31.4 (Pt^4+^) | 2.63 |
|  | 68.6 (Pt^2+^) |  |

For XPS analysis, the average oxidation state of Pt is calculated according to the below equation：

$$\begin{aligned} {Pt}^{\delta+}= Content\%\left( {Pt}^{4+} \right)\times4 + Content\%\left( {Pt}^{2+} \right)\times2 + Content\%\left( {Pt}^{0} \right)\times0 \end{aligned}$$

**Table S4** Potential of zero charge (PZC) calculated for different model systems

| Ads. form | $N_{{Pt}_{iso}}$ | $N_{{Pt}_{adj}}$ | $E_{\mathrm{pzc}}$(V) |
| --- | --- | --- | --- |
| MoS_2_ | 0 | 0 | 1.0638 |
| I | 1 | 0 | 0.8224 |
| II | 0 | 2 | 0.7597 |
| III | 2 | 0 | 0.5649 |
| IV | 1 | 2 | 0.5025 |

Note: The PZCs are presented as the potential *vs* SHE.

**Table S5** Extra charge injected into the electrode surface from external circuit at an electrode potential of 0 V *vs* SHE for different electrode materials

| Ads. form | $q_{\mathrm{ex}}$(e) |
| --- | --- |
|  |  |
| I | -1.1358 |
| II | -1.0909 |
| III | -0.8405 |
| IV | -0.7779 |

**Table S6** Hydrogen adsorption free energies of the most stable structures on Pt

| Adsorption site | $\Delta G_{ad}$(eV) | | | | |
| --- | --- | --- | --- | --- | --- |
|  | 1^st^ H | 2^nd^ H | 3^rd^ H | 4^th^ H | 5^th^ H |
| Pt_iso_ | -0.1426 | -0.1193 | -0.4594 | - | - |
| Pt_adj_ | -0.5616 | -0.0997 | -0.0722 or -0.0550 | 0.1811 | 0.1087 |

**Table S7** The adsorption energies of different H adsorption on Pt_iso_

| H number | Adsorption site | Energy | Total $E_{ad}$ (eV) | $E_{ad}$ per atom (eV) |
| --- | --- | --- | --- | --- |
| 0 | - | -244.6285 | - | - |
| 1 | a | -248.4863 | -0.3707 | -0.3707 |
| 1 | b | -248.2980 | -0.1825 | -0.1825 |
| 1 | c | -248.2982 | -0.1826 | -0.1826 |
| 2 | b, c | -252.3179 | -0.7153 | -0.3576 |
| 2 | a, c | -252.3230 | -0.7203 | -0.3602 |
| 2 | a, b | -252.3238 | -0.7211 | -0.3606 |
| 3 | a, b, c | -256.5369 | -1.4471 | -0.4824 |
| 4 | 2a, b, c | -259.2187 | -0.6418 | -0.1604 |
| 4 | a, 2b, c | -259.3157 | -0.7389 | -0.1847 |

**Table S8** Adsorption energies of different H adsorption on Pt_iso_ at different structures

| H number | Adsorption site | I | IV |
| --- | --- | --- | --- |
| 1 | a | -0.3707 | -0.3931 |
| 2 | a, b | -0.3504 | -0.3714 |
| 3 | a, b, c | -0.7260 | -0.7144 |

**Table S9** Vibrational energies and entropies for the most stable structure on Pt_iso_. *T*=298.15 *K*

| Ads. form | $N_{H}$@Pt_iso_ | $E_{\mathrm{zpe}}$(eV) | $E_{V}$(eV) | $S$(J/mol K) | $TS$(eV) | $G_{corr}$(eV) |
| --- | --- | --- | --- | --- | --- | --- |
| I | 0 | 0.1729 | 0.3464 | 110.4140 | 0.3412 | 0.0052 |
|  | 1 | 0.3751 | 0.5575 | 112.1344 | 0.3465 | 0.2110 |
|  | 2 | 0.5924 | 0.7842 | 117.9665 | 0.3645 | 0.4197 |
|  | 3 | 0.8394 | 1.0357 | 120.2911 | 0.3717 | 0.6640 |
| IV | 0 | 0.1736 | 0.3467 | 109.9675 | 0.3398 | 0.0068 |
|  | 1 | 0.3762 | 0.5575 | 111.8872 | 0.3457 | 0.2118 |
|  | 2 | 0.5891 | 0.7827 | 119.6924 | 0.3699 | 0.4128 |
|  | 3 | 0.8413 | 1.0355 | 118.5289 | 0.3663 | 0.6693 |

**Table S10** Adsorption energies of different H adsorption sites on Pt_adj_

| H number | Adsorption site | Energy | Total $E_{ad}$ (eV) | $E_{ad}$ per atom (eV) |
| --- | --- | --- | --- | --- |
| 0 | - | -248.5108 | - | - |
| 1 | a | -252.5213 | -0.5234 | -0.5234 |
| 1 | c | -252.7895 | -0.7915 | -0.7915 |
| 2 | c, e | -256.5853 | -1.1002 | -0.5501 |
| 2 | c, d | -252.5213 | -1.0195 | -0.5098 |
| 3 | c, d, e | -260.3675 | -1.3954 | -0.4651 |
| 3 | c, a, e | -260.3027 | -1.3305 | -0.4435 |
| 3 | c, b, e | -260.3689 | -1.3968 | -0.4656 |
| 4 | c, b, d, e | -264.2730 | -1.8138 | -0.4534 |
| 5 | c, a, b, d, e | -268.0981 | -2.1518 | -0.4304 |

**Table S11** Vibrational energies and entropies for some structures on Pt_adj_. *T*=298.15 K

| Number of  H@Pt_adj_ | Adsorption site | $E_{\mathrm{zpe}}$(eV) | $E_{V}$(eV) | $S$(J /mol K) | $TS$(eV) | $G_{corr}$(eV) |
| --- | --- | --- | --- | --- | --- | --- |
| 0 | - | 0.3000 | 0.6059 | 198.2921 | 0.6127 | -0.0069 |
| 1 | c | 0.4919 | 0.7983 | 193.3963 | 0.5976 | 0.2007 |
| 2 | c, e | 0.6886 | 1.0084 | 200.9849 | 0.6211 | 0.3874 |
| 3 | c, d, e | 0.9104 | 1.2390 | 205.1213 | 0.6338 | 0.6052 |
| 3 | c, a, e | 0.8902 | 1.2209 | 205.2180 | 0.6341 | 0.5868 |
| 3 | c, b, e | 0.8923 | 1.2218 | 204.6922 | 0.6325 | 0.5893 |
| 4 | c, b, d, e | 1.1097 | 1.4482 | 208.8082 | 0.6452 | 0.8030 |
| 5 | c, a, b, d, e | 1.3257 | 1.6747 | 215.1256 | 0.6648 | 1.0100 |

**Table S12** Adsorption energies of different H adsorption on aggregated Pt at different structures.

| H number | Adsorption site | II | IV | 3H@IV |
| --- | --- | --- | --- | --- |
| 1 | c | -0.7915 | -0.7233 | -0.7692 |
| 2 | c, e | -0.3087 | -0.3886 | -0.3311 |
| 3 | c, d, e | -0.2951 | -0.2548 | -0.2893 |
| 3 | c, a, e | -0.2303 | -0.2181 | -0.2259 |
| 3 | c, b, e | -0.2965 | -0.3142 | -0.2983 |
| 4 | c, b, d, e | -0.4170 | -0.4007 | -0.4100 |
| 5 | c, a, b, d, e | -0.3380 | -0.2837 | -0.3341 |

**Table S13** Adsorption energies of different H adsorption on Pt at different systems.

| Pt atom type | H number | Adsorption site | MoSe_2_ | WS_2_ | WSe_2_ |
| --- | --- | --- | --- | --- | --- |
|  | 1 | a | -0.3941 | -0.3945 | -0.4109 |
| Pt_iso_ | 2 | a, b | -0.3976 | -0.4624 | -0.4882 |
|  | 3 | a, b, c | -0.6046 | -0.7696 | -0.6212 |
|  | 1 | c | -0.7186 | -0.7985 | -0.7226 |
|  | 2 | c, e | -0.3221 | -0.3729 | -0.4038 |
| Pt_adj_ | 3 | c, d, e | -0.2392 | -0.2751 | -0.2326 |
|  | 3 | c, b, e | -0.3549 | -0.3949 | -0.4254 |
|  | 4 | c, b, d, e | -0.3025 | -0.3813 | -0.3018 |
|  | 5 | c, a, b, d, e | -0.3643 | -0.4033 | -0.4401 |

**Supplementary References**

1. J. Hempelmann, P.C. Müller, C. Ertural, R. Dronskowski, The orbital origins of chemical bonding in Ge−Sb−Te phase-change materials. Angew. Chem. Int. Ed. **61**(17), e202115778 (2022). <https://doi.org/10.1002/anie.202115778>
2. Q. Yang, H. Liu, P. Yuan, Y. Jia, L. Zhuang et al., Single carbon vacancy traps atomic platinum for hydrogen evolution catalysis. J. Am. Chem. Soc. **144**(5), 2171–2178 (2022). <https://doi.org/10.1021/jacs.1c10814>
3. D. Liu, X. Li, S. Chen, H. Yan, C. Wang et al., Atomically dispersed platinum supported on curved carbon supports for efficient electrocatalytic hydrogen evolution. Nat. Energy **4**(6), 512–518 (2019). <https://doi.org/10.1038/s41560-019-0402-6>
4. H. Zhang, P. An, W. Zhou, B.Y. Guan, P. Zhang et al., Dynamic traction of lattice-confined platinum atoms into mesoporous carbon matrix for hydrogen evolution reaction. Sci. Adv. **4**(1), eaao6657 (2018). <https://doi.org/10.1126/sciadv.aao6657>
5. Z. Zhang, Y. Chen, L. Zhou, C. Chen, Z. Han et al., The simplest construction of single-site catalysts by the synergism of micropore trapping and nitrogen anchoring. Nat. Commun. **10**, 1657 (2019). <https://doi.org/10.1038/s41467-019-09596-x>
6. T. Li, J. Liu, Y. Song, F. Wang, Photochemical solid-phase synthesis of platinum single atoms on nitrogen-doped carbon with high loading as bifunctional catalysts for hydrogen evolution and oxygen reduction reactions. ACS Catal. **8**(9), 8450–8458 (2018). <https://doi.org/10.1021/acscatal.8b02288>
7. Q. Cheng, C. Hu, G. Wang, Z. Zou, H. Yang et al., Carbon-defect-driven electroless deposition of Pt atomic clusters for highly efficient hydrogen evolution. J. Am. Chem. Soc. **142**(12), 5594–5601 (2020). <https://doi.org/10.1021/jacs.9b11524>
8. X. Cheng, Y. Lu, L. Zheng, Y. Cui, M. Niibe et al., Charge redistribution within platinum–nitrogen coordination structure to boost hydrogen evolution. Nano Energy **73**, 104739 (2020). <https://doi.org/10.1016/j.nanoen.2020.104739>
9. X.-P. Yin, H.-J. Wang, S.-F. Tang, X.-L. Lu, M. Shu et al., Engineering the coordination environment of single-atom platinum anchored on graphdiyne for optimizing electrocatalytic hydrogen evolution. Angew. Chem. Int. Ed. **57**(30), 9382–9386 (2018). <https://doi.org/10.1002/anie.201804817>
10. D. Cao, H. Xu, H. Li, C. Feng, J. Zeng et al., Volcano-type relationship between oxidation states and catalytic activity of single-atom catalysts towards hydrogen evolution. Nat. Commun. **13**, 5843 (2022). <https://doi.org/10.1038/s41467-022-33589-y>
11. N. Cheng, S. Stambula, D. Wang, M.N. Banis, J. Liu et al., Platinum single-atom and cluster catalysis of the hydrogen evolution reaction. Nat. Commun. **7**, 13638 (2016). <https://doi.org/10.1038/ncomms13638>
12. P. Kuang, Y. Wang, B. Zhu, F. Xia, C.-W. Tung et al., Pt single atoms supported on N-doped mesoporous hollow carbon spheres with enhanced electrocatalytic H2-evolution activity. Adv. Mater. **33**(18), 2008599 (2021). <https://doi.org/10.1002/adma.202008599>
13. T. Ma, H. Cao, S. Li, S. Cao, Z. Zhao et al., Crystalline lattice-confined atomic Pt in metal carbides to match electronic structures and hydrogen evolution behaviors of platinum (adv. mater. 41/2022). Adv. Mater. **34**(41), 2270284 (2022). <https://doi.org/10.1002/adma.202270284>
14. S. Ye, F. Luo, Q. Zhang, P. Zhang, T. Xu et al., Highly stable single Pt atomic sites anchored on aniline-stacked graphene for hydrogen evolution reaction. Energy Environ. Sci. **12**(3), 1000–1007 (2019). <https://doi.org/10.1039/c8ee02888e>
15. H. Wei, H. Wu, K. Huang, B. Ge, J. Ma et al., Ultralow-temperature photochemical synthesis of atomically dispersed Pt catalysts for the hydrogen evolution reaction. Chem. Sci. **10**(9), 2830–2836 (2019). <https://doi.org/10.1039/c8sc04986f>
16. R. Li, J. Xu, Q. Zhao, W. Ren, R. Zeng et al., Cathodic corrosion as a facile and universal method for the preparation of supported metal single atoms. Nano Res. **15**(3), 1838–1844 (2022). <https://doi.org/10.1007/s12274-021-3767-3>
17. Z. Zhang, Z. Zhang, C. Chen, R. Wang, M. Xie et al., Single-atom platinum with asymmetric coordination environment on fully conjugated covalent organic framework for efficient electrocatalysis. Nat. Commun. **15**, 2556 (2024). <https://doi.org/10.1038/s41467-024-46872-x>
18. S. Fang, X. Zhu, X. Liu, J. Gu, W. Liu et al., Uncovering near-free platinum single-atom dynamics during electrochemical hydrogen evolution reaction. Nat. Commun. **11**, 1029 (2020). <https://doi.org/10.1038/s41467-020-14848-2>
19. X. Cheng, B. Xiao, Y. Chen, Y. Wang, L. Zheng et al., Ligand charge donation–acquisition balance: a unique strategy to boost single Pt atom catalyst mass activity toward the hydrogen evolution reaction. ACS Catal. **12**(10), 5970–5978 (2022). <https://doi.org/10.1021/acscatal.2c00891>
20. J. Xu, R. Li, R. Zeng, X. Yan, Q. Zhao et al., Platinum single atoms supported on nanoarray-structured nitrogen-doped graphite foil with enhanced catalytic performance for hydrogen evolution reaction. ACS Appl. Mater. Interfaces **12**(34), 38106–38112 (2020). <https://doi.org/10.1021/acsami.0c09615>
21. C. Li, Z. Chen, H. Yi, Y. Cao, L. Du et al., Polyvinylpyrrolidone-coordinated single-site platinum catalyst exhibits high activity for hydrogen evolution reaction. Angew. Chem. Int. Ed. **59**(37), 15902–15907 (2020). <https://doi.org/10.1002/anie.202005282>
22. S. Zhang, Z. Wu, Y. Liu, J. Bai, Y. Ding et al., Single-atom platinum immobilized on polyimide for highly efficient and durable hydrogen evolution electrocatalysis. Adv. Energy Mater. **15**(17), 2403945 (2025). <https://doi.org/10.1002/aenm.202403945>
23. X. Cheng, Y. Lu, L. Zheng, Y. Cui, M. Niibe et al., Charge redistribution within platinum–nitrogen coordination structure to boost hydrogen evolution. Nano Energy **73**, 104739 (2020). <https://doi.org/10.1016/j.nanoen.2020.104739>
24. S. Jiao, M. Kong, Z. Hu, S. Zhou, X. Xu et al., Pt atom on the wall of atomic layer deposition (ALD)-made MoS_2_ nanotubes for efficient hydrogen evolution. Small **18**(16), 2105129 (2022). <https://doi.org/10.1002/smll.202105129>
25. C. Yue, C. Feng, G. Sun, N. Liu, H. Hao et al., Hierarchically stabilized Pt single-atom catalysts induced by an atomic substitution strategy for an efficient hydrogen evolution reaction. Energy Environ. Sci. **17**(14), 5227–5240 (2024). <https://doi.org/10.1039/D3EE04457B>
26. K. Jiang, B. Liu, M. Luo, S. Ning, M. Peng et al., Single platinum atoms embedded in nanoporous cobalt selenide as electrocatalyst for accelerating hydrogen evolution reaction. Nat. Commun. **10**, 1743 (2019). <https://doi.org/10.1038/s41467-019-09765-y>
27. J. Zhang, E. Wang, S. Cui, S. Yang, X. Zou et al., Single-atom Pt anchored on oxygen vacancy of monolayer Ti_3_C_2_T*_x_* for superior hydrogen evolution. Nano Lett. **22**(3), 1398–1405 (2022). <https://doi.org/10.1021/acs.nanolett.1c04809>
28. L. Liu, Y. Wang, Y. Zhao, Y. Wang, Z. Zhang et al., Ultrahigh Pt-mass-activity hydrogen evolution catalyst electrodeposited from bulk Pt. Adv. Funct. Mater. **32**(20), 2112207 (2022). <https://doi.org/10.1002/adfm.202112207>
29. Y. Shi, Z.-R. Ma, Y.-Y. Xiao, Y.-C. Yin, W.-M. Huang et al., Electronic metal–support interaction modulates single-atom platinum catalysis for hydrogen evolution reaction. Nat. Commun. **12**, 3021 (2021). <https://doi.org/10.1038/s41467-021-23306-6>
30. M. Sheng, X. Bin, Y. Yang, Y. Tang, W. Que, *In situ* electrosynthesis of MAX-derived electrocatalysts for superior hydrogen evolution reaction. Small **18**(32), 2203471 (2022). <https://doi.org/10.1002/smll.202203471>
31. H. Yuan, D. Jiang, Z. Li, X. Liu, Z. Tang et al., Laser synthesis of PtMo single-atom alloy electrode for ultralow voltage hydrogen generation. Adv. Mater. **36**(5), 2305375 (2024). <https://doi.org/10.1002/adma.202305375>
32. S.J. Park, T.H. Nguyen, D.T. Tran, V.A. Dinh, J.H. Lee et al., Delaminated MBene sheets beyond usual 2D transition metal materials for securing Pt single atoms to boost hydrogen evolution. Energy Environ. Sci. **16**(9), 4093–4104 (2023). <https://doi.org/10.1039/D3EE01314F>
33. J. Zhu, Y. Tu, L. Cai, H. Ma, Y. Chai et al., Defect-assisted anchoring of Pt single atoms on MoS_2_ nanosheets produces high-performance catalyst for industrial hydrogen evolution reaction. Small **18**(4), 2104824 (2022). <https://doi.org/10.1002/smll.202104824>
